# Electrosynthesis of polycyclic quinazolinones and rutaecarpine from isatoic anhydrides and cyclic amines†

**DOI:** 10.1039/d0ra09382c

**Published:** 2020-12-16

**Authors:** Xingyu Chen, Xing Zhang, Sixian Lu, Peng Sun

**Affiliations:** Institute of Chinese Meteria Medica, Artermisinin Research Center, Academy of Chinese Medical Sciences Beijing 100700 P. R. China psun@icmm.ac.cn

## Abstract

A direct decarboxylative cyclization between readily available isatoic anhydrides and cyclic amines was established to construct polycyclic fused quinazolinones employing electrochemical methods. This procedure was performed in an undivided cell without the use of a transition-metal-catalyst and external oxidant. A broad scope of polycyclic fused quinazolinones were obtained in moderate to good yields. Additionally, rutaecarpine was also prepared through our method in one step in good yield.

## Abstract 2

Polycyclic quinazolinones and rutaecarpine were synthesized from isatoic anhydrides and cyclic amines through an electrochemical method without an external oxidant and transition-metal-catalyst.

## Graphical abstract

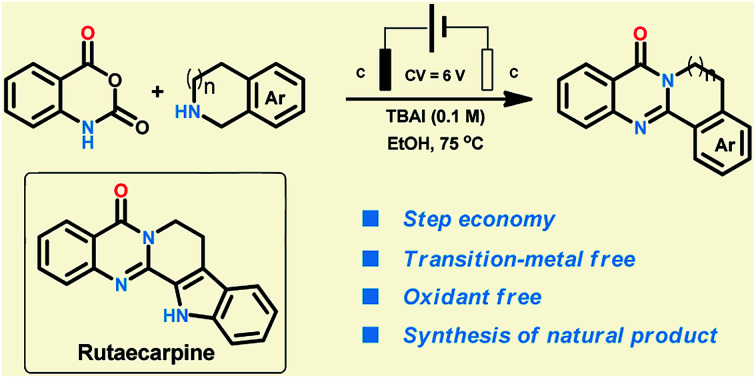

Quinazolinones represent a class of important nitrogen containing heterocyclic compounds which are widely distributed in natural products and synthetic materials. Over the last few decades, hundreds of quinazolinone alkaloids with a polycyclic structural motif have been discovered and studied for various purposes.^1^ Representatively, rutaecarpine and its derivatives attracted considerable attention of pharmaceutical scientists due to their broad biological activities including antiobesity,^2^ anti-tumor,^3^ and cardiovascular protection.^4^ Owing to their wide applications, tremendous efforts have been devoted to the construction of this privileged skeleton. The classical synthetic approaches^5^ to quinazolinones depend on the condensation between 2-aminobenzoic acid derivatives and other synthons, which suffered from tedious steps, hash conditions, limited scope and poor yields. To overcome these shortcomings, a number of methodologies were established to synthesis polycyclic quinazolinones. Thanks to the rapid development of transition-metal catalyzed C–H activation reactions, Pd,^6^ Ru,^7^ and Rh^8^ catalyzed intermolecular cyclization reactions between pre-synthesized quinazolinones and alkynes, vinyl/allyl acetates, sulfoxonium ylides were developed successively to prepare poly-cyclic quinazolinones (Scheme 2a). Alternatively, Zheng and co-workers explored light^9^ or (NH_4_)_2_S_2_O_8_ (ref. 10) induced intramolecular cyclization of 2-aminobenzamide and 2-nitrobenzamide derivatives, the products were synthesized under metal-free conditions (Scheme 2b). These brilliant works provide us efficient routes to polycyclic quinazolinones, however, the highly-functionalized substrates they used need to be synthesized in advance in several steps. More convenient and straightforward synthetic methods to produce polycyclic quinazolinones from readily available materials are still in need. Intermolecular oxidative annulation reaction using cyclic amines were reported by Zhang^11^ and Li^12^ to offer polycyclic fused quinazolinones employing oxygen as oxidant (Scheme 2c). Very recently, TBHP (*t*-butyl hydroperoxide) mediated reaction between isatins and tetrahydroisoquinolines *via* cascade Baeyer–Villiger oxidation rearrangement and cyclization were achieved by us^13^ and Hu^14^ respectively. In the last few years, the prosperity of electrochemistry provided us green and economy instrument for organic synthesis.^15^ Isatoic anhydrides are versatile bulk chemical materials which have been used to synthesis a large variety of compound especially N-heterocycles.^16^ Herein, as our continuous interests in the construction of bioactive heterocycles,^17^ we reported a direct electrochemical intermolecular [4 + 2] cycloaddition between readily available isatoic anhydrides and cyclic amines leading to polycyclic quinazolinones and rutaecarpine (Scheme 2d). This protocol exhibited mild conditions, excellent yield and good functional group tolerance. No additional oxidant and transition-metal-catalyst was need in the overall process (Scheme 1).

**Scheme 1:**
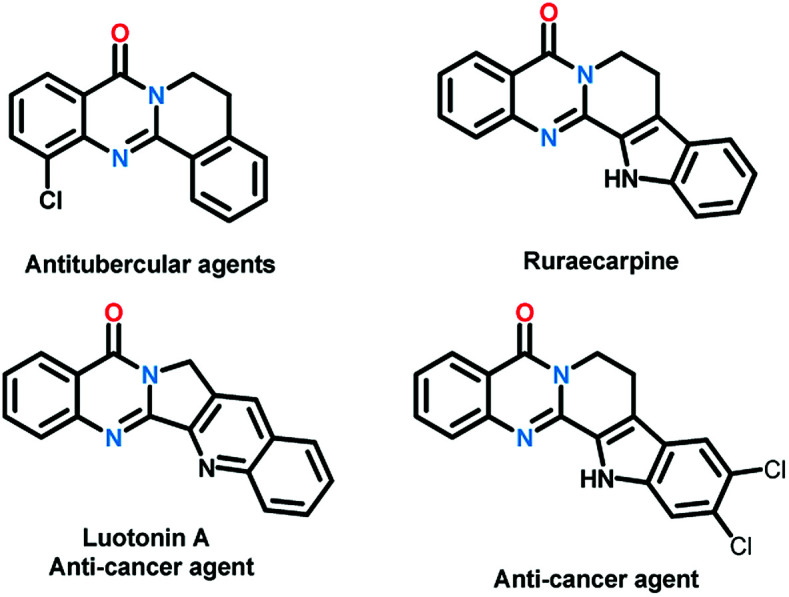
Bioactive polycyclic quinazolinones.

**Scheme 2:**
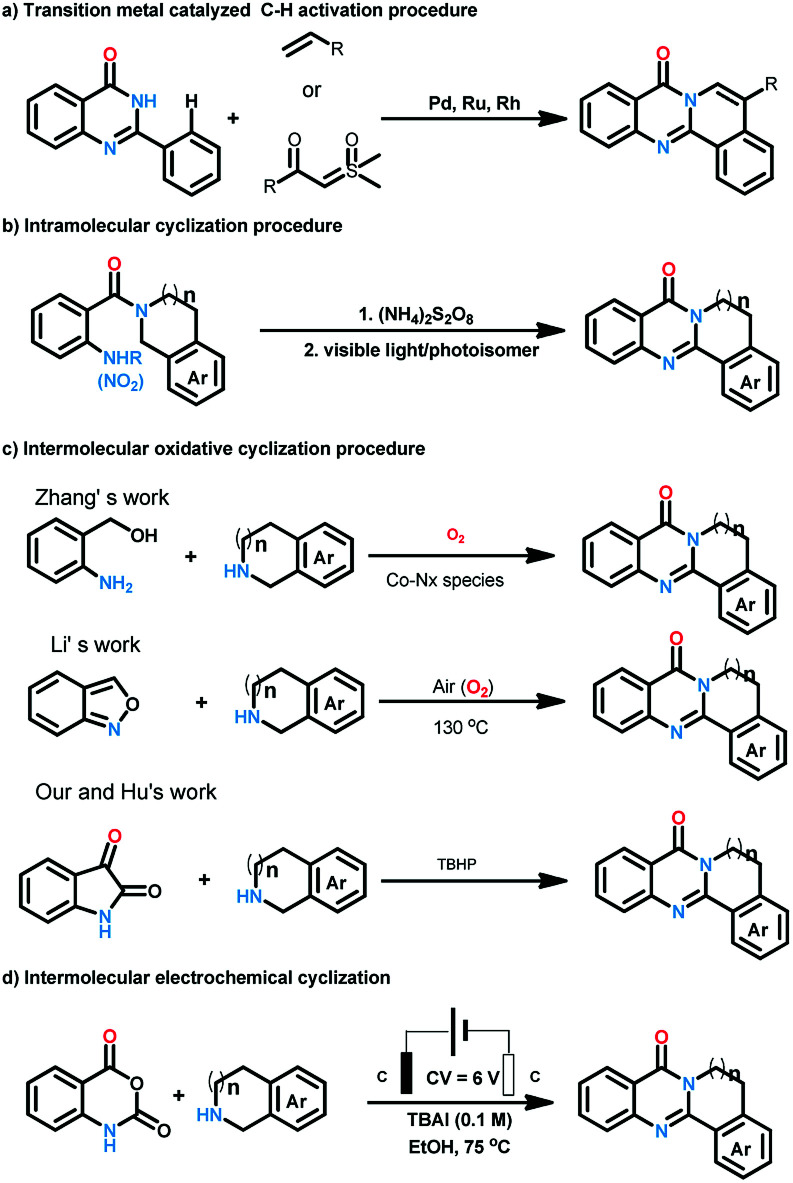
Synthesis of polycyclic quinazolinones.

We start our investigation with isatoic anhydride (1a) and tetrahydroisoquinoline (2a) as the model substrates. The reaction was conducted in an undivided cell equipped with two graphite electrodes. At first, a series of commonly used electrolytes including perchlorate and ammonium were evaluated. There was no de-sired product generated until potassium iodide was employed as electrolyte (entries 1–5). When potassium iodide was replaced with tetra-butyl ammonium iodide (TBAI), a moderate yield of 66% was obtained (entry 6). Later, DMF was examined to maintain the yield to 72% (entry 7). The yield was promoted to over 80% when protic solvent such as MeOH, EtOH, TFE and HFIP were used (entries 8–11). It was worth mentioning that an excellent yield of 88% was obtained when the reaction was performed in EtOH (entry 9). Only 32% product was generated in H_2_O may be attributed to the poor solubility of the reactant (entry 12). The yield was obviously decreased to 45% with the reduction of the temperature to 50 °C (entry 13). The reaction did not occur at room temperature (entry 14) (Table 1).

**Table 1:** Optimization of reaction conditions^a^

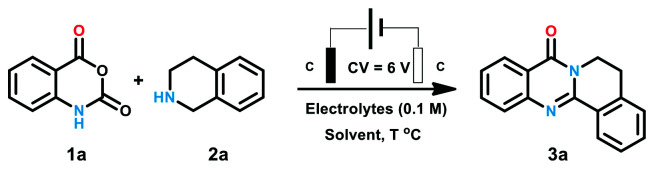				
Entry	Solvent	Electrolyte	Temperature	Yield (3a)^b^
1	EtOH	NaClO_4_	75 °C	0%
2	CH_3_CN	LiClO_4_	75 °C	0%
3	CH_3_CN	*n*Bn_4_NBF_4_	75 °C	0%
4	CH_3_CN	*n*Bn_4_NPF_6_	75 °C	0%
5	CH_3_CN	KI	75 °C	33%
6	CH_3_CN	TBAI	75 °C	66%
7	DMF	TBAI	75 °C	72%
8	MeOH	TBAI	75 °C	80%
9	EtOH	TBAI	75 °C	88%
10	TFE	TBAI	75 °C	85%
11	HFIP	TBAI	75 °C	82%
12	H_2_O	TBAI	75 °C	32%
13	EtOH	TBAI	50 °C	45%
14	EtOH	TBAI	25 °C	0%

a Reaction conditions: 1a (1.2 mmol, 1.2 equiv.), 2a (1.0 mmol, 1.0 equiv.), electrolyte (1.0 M), solvent (10 mL), in an undivided cell equipped with two graphite electrodes, constant voltage (6.0 V) for 6 hours under air atmosphere.

b Isolated yields.

Based on the optimized conditions, we turned to explore the scope of the reaction. We are pleased to find that this procedure exhibited excellent functional group tolerance, as shown in Table 2. Firstly, a wide range of isatoic anhydride derivatives were investigated to be compatible with the reaction conditions. Isatoic anhydride with methyl at C-8 position delivered products 3b in the yield of 81%. Substrate with methoxyl and bromine at C-7 position were also proper for the optimized conditions and corresponding product 3c and 3d was prepared in 92% and 79% yields. Next, a series of isatoic anhydride with different electronic effect groups at C6-position of the benzene ring were tested. Electron neutral and donating groups (6-Me and 6-OMe) performed well to yield 3e and 3f in high yields (90% and 92%, respectively). When 6-trifluoromethoxy isatoic anhydride was tested, the expected product 3g was also obtained in the yield of 88%. Additionally, a battery of halogen-substituted isatoic anhydrides at the C-6 position of benzene ring were also proved to be favorable for this electrochemical transformation, and the corresponding products (3h–3j) were synthesized in the yield over 75%. According to our previous research, the product with iodine at the benzene ring could be transformed to structural diverse compounds through Pd catalyzed cross coupling reaction with alkyne, aryl-boronic acid and amines. Isatoic anhydride with strong electron withdrawing NO_2_ failed to give corresponding products. Then, products with substituent group at C-5 position of the benzene ring were also generated in the yields of 79% to 81% (3k–3l). We also examined the multi-substituted isatoic anhydrides to provided structural diverse products in the yield of 69% (3m). We then turned our attention to the reaction with respect to the amines. A series of tetrahydroisoquinoline was loaded to the reaction system and desired products were generated in good yields. Either electron donating methoxyl group (3o) or electron withdrawing nitro group (3q) was proved to be tolerable for the process. These results indicated that the electronic characters of the benzene ring of tetrahydroisoquinoline has no obvious influence on the reaction outcomes. We are pleased to find that di-substituted quinazolinones 3r was generated in 89% yield. Apart from tetrahydroisoquinolines, isoindoline with five-membered cyclic amines was also examined to generate 3s in 81% yield.

**Table 2:** Scope of the reaction^a^^,^^b^

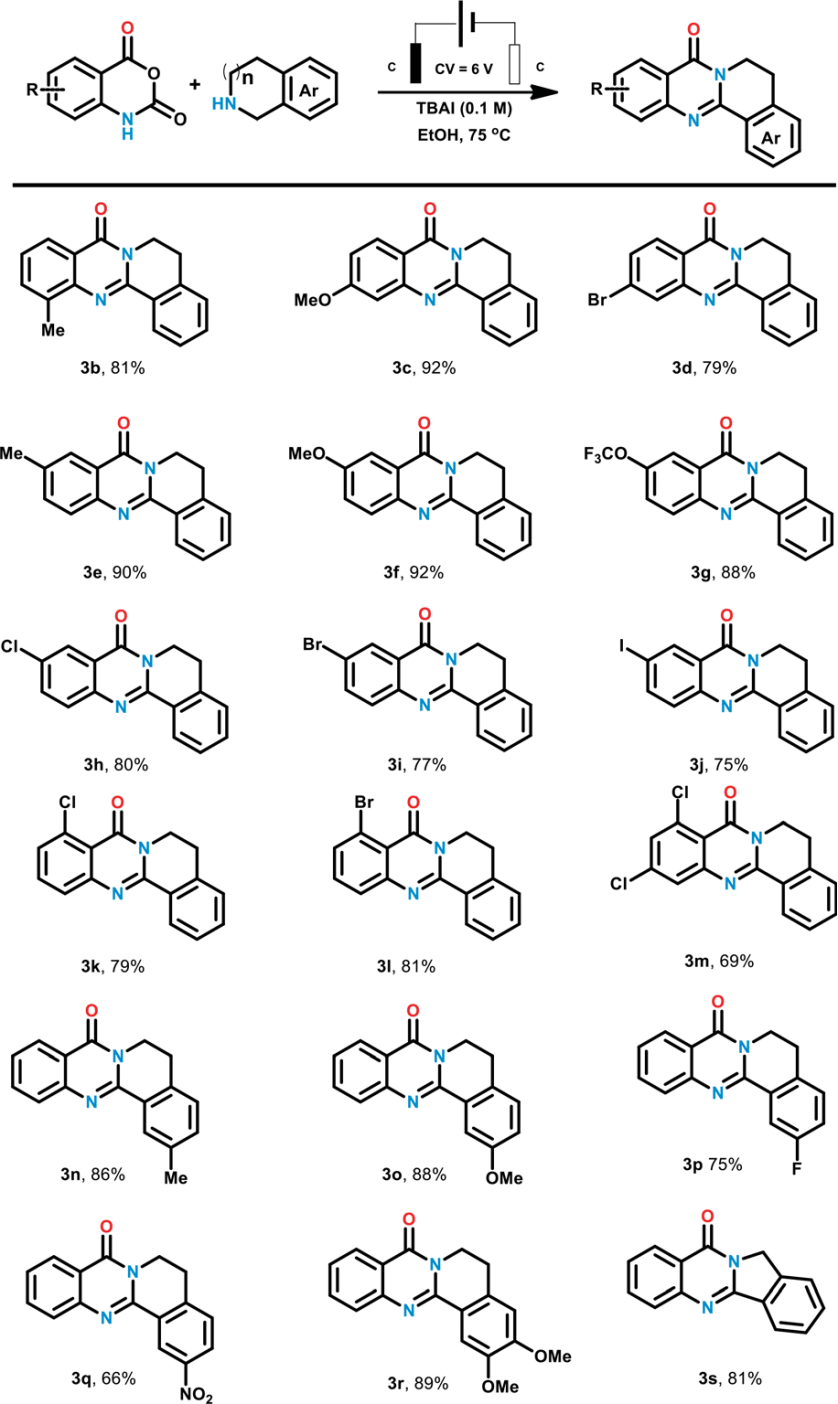

a Reaction conditions: 1a (1.2 mmol, 1.2 equiv.), 2a (1.0 mmol, 1.0 equiv.), TBAI (1.0 M), EtOH (10 mL), in an undivided cell equipped with two graphite electrodes, constant voltage (6.0 V) for 6 hours under air atmosphere.

b Isolated yields.

After the substrates scope exploration, the reaction was amplified to 10 mmol scales to illustrate the synthetic applications of the process. To our delight, this electrochemical procedure performed smoothly at gram scale to afford the product in 69% yield (Scheme 3a). Rutaecarpine is an interesting natural product with polycyclic quinazolinone skeleton. The convenient and environmental benign synthetic method for bioactive natural products is one of the most pursued issues for chemists. Herein, rutaecarpine was synthesised through our method from commercially available starting materials in the yield of 80% (Scheme 3b).

**Scheme 3:**
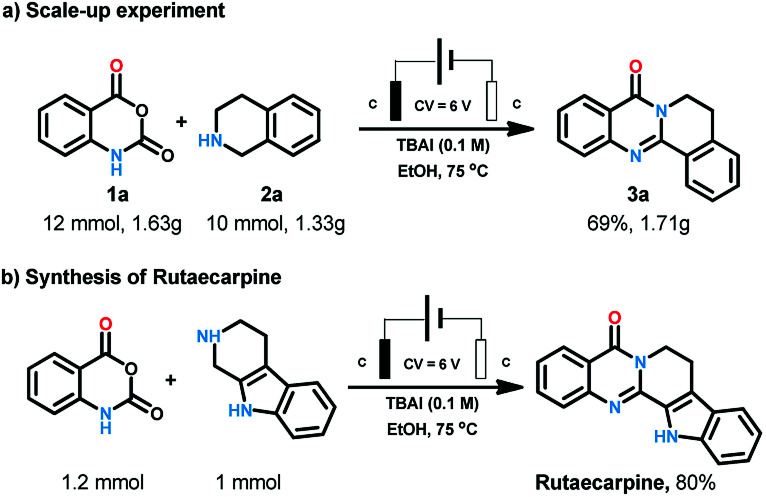
Synthetic application of the reaction.

In the course of condition screening we found that only iodine anion electrolyte could generate the desired product. Molecular iodine and iodine reagents-mediated reactions have been well explored as powerful instruments for organic synthesis due to their characters of cheap, green, and easily available.^18^ When we replaced the electrochemical conditions with iodine, 80% yield was also obtained (Scheme 4). When radical scavengers such as TEMPO (2,2,6,6-tetramethylpiperidine-1-oxyl) and BHT (butylated hydroxytoluene) (Scheme 4) were loaded to the reaction system, the yield were reduced to less than 30%. When 2a was treated with the standard conditions for two hours, 3,4-dihydroisoquinoline (4) was obtained in the yield of 81%. The combination of 1a and 4 offer 3a in the yield of 98% under standard conditions. When 1a and 2a were heated at 75 °C for 12 hours, 5 was generated in 95% yield. We treated 5 under standard conditions for 12 hours, 27% 3a was obtained with 70% starting material recovered.

**Scheme 4:**
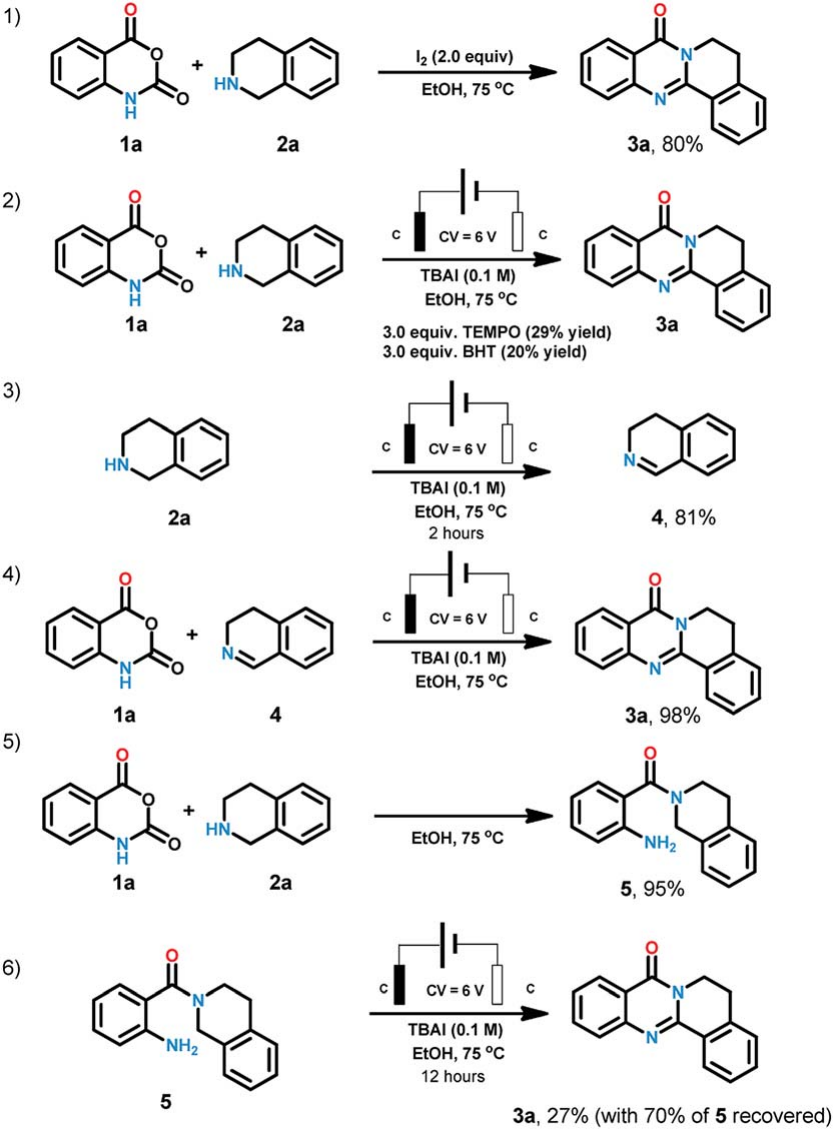
Mechanism study.

The cyclic voltammetry experiments have been conducted to determine the redox potential of the starting material (for details see ESI†). Based on these results and previous reports, we proposed the plausible reaction pathway as shown in Scheme 5. The reaction could be accomplished in two paths. Firstly, the iodine anion was oxidized at anode to generate iodine radical which subsequently offered iodine. Next, intermediate 4 produced *via* the oxidation of 2a by iodine with the elimination of HI or direct anodic oxidation. The condensation of 1a and 4 led to intermediate 6. Finally, the oxidation of compound 6 under the function of iodine^19^ or anode offered the desired products 3a. Alternatively, the desired product could also be generated through another pathway. At first, the reactions between 1a and 2a yielded intermediate 5, which could be transformed to cation radical intermediate 5′ and 7 through successive anodic oxidation processes. The following intermolecular nucleophilic cyclization afforded 6. Then 3a was produced through the oxidation of 6 as same as path A.

**Scheme 5:**
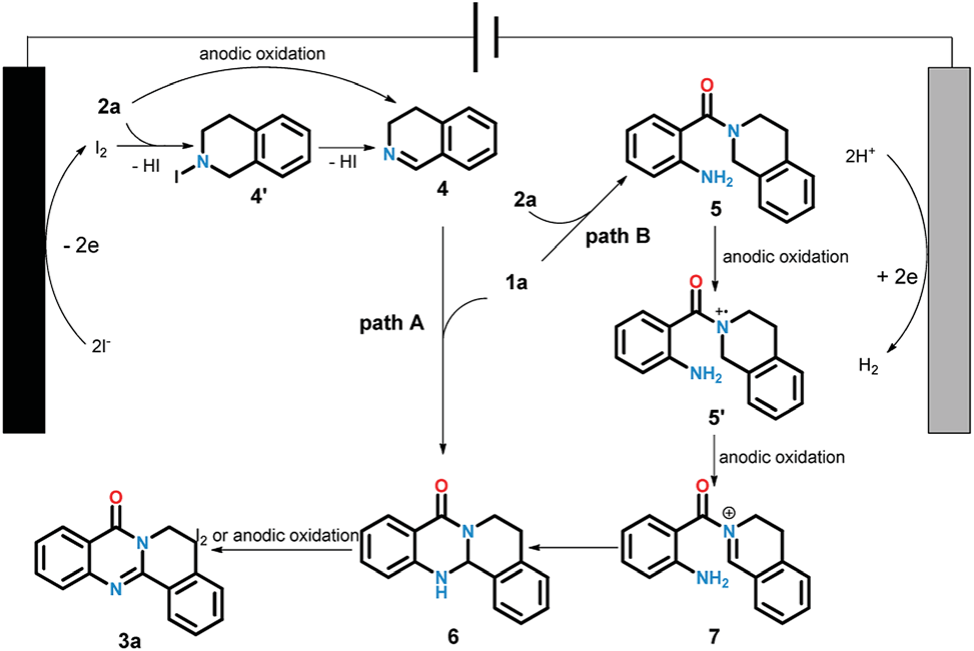
Proposed reaction pathway.

## Conclusions

In summary, we have developed a straightforward electro-chemical approach to construct polycyclic quinazolinones from readily available isatoic anhydrides and cyclic amines in one step. A broad scope of fused quinazolinones were prepared in moderate to good yield in the absence of transition-metal catalyst and external oxidant. This method also provided efficient route to rutaecarpine.

## Conflicts of interest

There are no conflicts to declare.

## Supplementary Material

RA-010-D0RA09382C-s001
