# Supplementary material for: Electrosynthesis of polycyclic quinazolinones and rutaecarpine from isatoic anhydrides and cyclic amines
Source: RSC Adv. 2020 Dec 16;10(72):44382–6. doi: 10.1039/d0ra09382c (PMC9058480; doi:10.1039/d0ra09382c)
Supplement: RA-010-D0RA09382C-s001 [file RA-010-D0RA09382C-s001.pdf]

Electronic Supplementary Material (ESI) for RSC Advances.
This journal is © The Royal Society of Chemistry 2020

Supporting Information

Electrosynthesis of Polycyclic Quinazolinones and Rutaecarpine from Isatoic Anhydrides and Cyclic Amines

Xingyu Chen,# Xing Zhang,# Sixian Lu, Peng Sun.*

Institute of Chinese Materia Medica and Artemisinin Research Center, China Academy of Chinese Medical Sciences Beijing 100700, P. R. China.

Email : psun@icmm.ac.cn

Contents

1.General Informations **..S2**

2.General Procedure for The Electrosynthesis of Polycyclic Quinazolinones **..S2**

3. Cyclic Voltammetry experiments **...S12**

4.*^{1}H NMR and ^{13}C NMR of the Products* **....................................S13**

1. General Informations

All the starting materials and solvents were commercially available and were used without further purification unless otherwise stated. All reactions were carried out in an undivided cell equipped with two graphite electrodes. The ^{1}H and ^{13}C NMR were recorded on Bruker-AV 600 spectrometer and chemical shifts reported in $CDCl_3$ or DMSO-$_{d6}$ with tetramethylsilane as an internal standard. High Resolution Mass measurement was performed on Waters QTOF 6520 mass spectrometer with electron spray ionization (ESI) as the ion source. Melting point (mp) was measured on a microscopic melting point apparatus. The reactions were performed in an undivided cell equipped with two graphite electrodes, and the electrochemical conditions were provided by the Electrasyn 2.0 from IKA as shown in Figure 1.

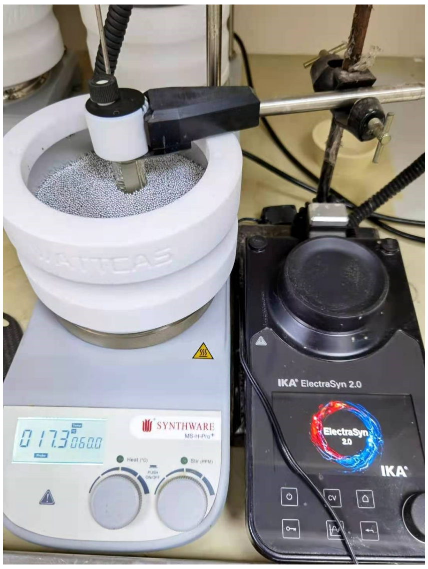

Figure 1. Picture of the reaction device

2. General Procedure for The Electrosynthesis of Polycyclic Quinazolinones

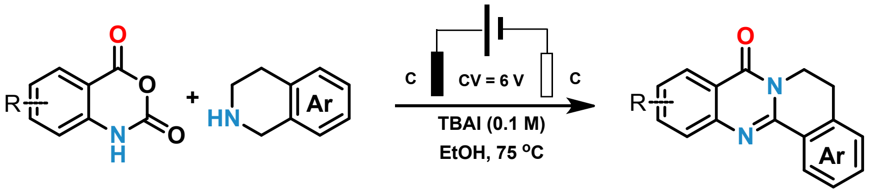

Conditions:

An undivided cell equipped with two graphite electrodes bottom flask was charged with isatoics anhydride **1** (1.2 mmol, 1.2 equiv.), cyclic amine **2** (1.0 mmol, 1.0 equiv.), TBAI (1mmol) and 10 mL EtOH. The reaction mixture was vigorously stirred under constant voltage 6.0 v at 75 oC for 6 hours. After cooling to room temperature, the reaction mixture was quenched with 10 mL of saturated sodium sulfite solution. Then extracted with ethyl acetate (20 mL) for two times. The organic phase was washed with water and brine respectively. The solvent was concentrated in vacuo and purified by flash chromatography on silica gel to afford the desired product.

Characterization of the Products:

5H-isoquinolino[1,2-b]quinazolin-8(6H)-one (3a)

Yield 88%; White solid, m.p. 193-195 °C; ^{1}H NMR (600 MHz, $CDCl_3$) δ 8.40 (d, J = 7.8 Hz, 1H), 7.63 (d, J = 2.1 Hz, 1H), 7.49 (td, J = 7.4, 1.4 Hz, 1H), 7.42 (t, J = 7.6 Hz, 1H), 7.40 (d, J = 2.1 Hz, 1H), 7.28 (d, J = 7.5 Hz, 1H), 4.33 (t, J = 6.5 Hz, 2H), 3.09 (t, J = 6.6 Hz, 2H). ppm; ^{13}C NMR (150 MHz, $CDCl_3$) δ 161.7, 149.4, 147.8, 137.1, 134.2, 131.7,

129.6, 128.0, 127.6, 127.5, 126.9, 126.5, 120.8, 100.0, 39.6, 27.5 ppm; HRMS (ESI) calcd for $[C_{16}H_{12}N_2O+H]^+$ 249.0950, found 249.1062.

12-methyl-5H-isoquinolino[1,2-b]quinazolin-8(6H)-one (3b)

Yield 81%; white solid, m.p. 165-167 °C; ^{1}H NMR ^{1}H NMR (600 MHz, $CDCl_3$) δ 8.52 (dd, *J* = 7.7, 1.6 Hz, 1H), 8.15 (dd, *J* = 8.0, 1.5 Hz, 1H), 7.58 (d, *J* = 7.2 Hz, 1H), 7.46 (td, *J* = 7.3, 1.6 Hz, 1H), 7.42 (td, *J* = 7.5, 1.4 Hz, 1H), 7.33 (t, *J* = 7.6 Hz, 1H), 7.28 – 7.24 (m, 1H), 4.40 (t, *J* = 6.5 Hz, 2H), 3.09 (t, *J* = 6.5 Hz, 2H), 2.70 (s, 3H) ppm; ^{13}C NMR (150 MHz, $CDCl_3$) δ 162.1, 148.0, 146.3, 136.9, 136.1, 134.7, 131.5, 130.0, 128.0, 127.6, 127.5, 126.1, 124.5, 120.7, 39.5, 27.5, 17.3 ppm; HRMS (ESI) calcd for $[C_{17}H_{14}N_2O+H]^+$ 263.1179, found 263.1175.

11-methoxy-5H-isoquinolino[1,2-b]quinazolin-8(6H)-one (3c)

Yield 92%; light yellow solid, m.p. 177-179 °C; ^{1}H NMR (600 MHz, $CDCl_3$) δ 8.46 (dd, *J* = 7.7, 1.4 Hz, 1H), 8.20 (d, *J* = 8.8 Hz, 1H), 7.48 (td, *J* = 7.4, 1.5 Hz, 1H), 7.46 – 7.39 (m, 1H), 7.29 (d, *J* = 7.4 Hz, 1H), 7.15 (d, *J* = 2.5 Hz, 1H), 7.04 (dd, *J* = 8.8, 2.5 Hz, 1H), 4.39 (t, *J* = 6.5 Hz, 2H), 3.94 (s, 3H), 3.09 (t, *J* = 6.5 Hz, 2H) ppm; ^{13}C NMR (150 MHz, $CDCl_3$) δ 164.5, 161.2, 150.0, 150.0, 137.1, 131.7, 129.6, 128.4, 127.9, 127.6, 127.5, 116.8, 114.4, 108.0, 55.6, 39.4, 27.5 ppm; HRMS (ESI) calcd for $[C_{17}H_{14}N_2O_2+H]^+$ 279.1128, found 279.1125.

11-bromo-5H-isoquinolino[1,2-b]quinazolin-8(6H)-one (3d)

Yield 79%; white solid, m.p. 176-178 °C; ^{1}H NMR (600 MHz, $CDCl_3$)

δ 8.43 (d, *J* = 7.8 Hz, 1H), 8.40 (d, *J* = 2.3 Hz, 1H), 7.79 (dd, *J* = 8.7, 2.3 Hz, 1H), 7.60 (d, *J* = 8.6 Hz, 1H), 7.47 (td, *J* = 7.4, 1.4 Hz, 1H), 7.42 (t, *J* = 7.5 Hz, 1H), 7.27 (d, *J* = 7.0 Hz, 1H), 4.38 (t, *J* = 6.5 Hz, 2H), 3.09 (t, *J* = 6.5 Hz, 2H) ppm; ^{13}C NMR (150 MHz, $CDCl_3$) ^{13}C NMR (150 MHz, $CDCl_3$) δ 161.2, 150.4, 148.8, 137.1, 132.0, 130.2, 129.8, 129.2, 128.8, 128.3, 128.2, 127.7, 127.5, 119.5, 39.6, 27.3 ppm; HRMS (ESI) calcd for $[C_{16}H_{11}BrN_2O+H]^+$ 327.0128, found 327.0127.

10-methyl-5H-isoquinolino[1,2-b]quinazolin-8(6H)-one (3e)

Yield 90%; White solid, m.p. 182-183 ^{o}C; ^{1}H NMR (600 MHz, $CDCl_3$) δ 8.46 (dd, *J* = 7.7, 1.5 Hz, 1H), 8.09 (d, *J* = 2.0 Hz, 1H), 7.66 (d, *J* = 8.2 Hz, 1H), 7.56 (dd, *J* = 8.4, 2.0 Hz, 1H), 7.43 (dtd, *J* = 21.9, 7.5, 1.4 Hz, 2H), 7.27 (d, *J* = 7.2 Hz, 1H), 4.40 (t, *J* = 6.4 Hz, 2H), 3.09 (t, *J* = 6.5 Hz, 2H), 2.49 (s, 3H) ppm; ^{13}C NMR (150 MHz, $CDCl_3$) δ 161.7, 148.6, 145.8, 136.9, 136.7, 135.7, 131.5, 129.7, 127.9, 127.6, 127.5, 127.4, 126.3, 120.5, 39.6, 27.5, 21.4 ppm; HRMS (ESI) calcd for $[C_{17}H_{14}N_2O+H]^+$ 263.1179, found 263.1176.

10-methoxy-5H-isoquinolino[1,2-b]quinazolin-8(6H)-one (3f)

Yield 92%; Light yellow solid, m.p. 169-170 ^{o}C; ^{1}H NMR (300 MHz, $CDCl_3$) δ 8.45 (dd, *J* = 7.6, 1.5 Hz, 1H), 7.71 (d, *J* = 8.9 Hz, 1H), 7.68 (d, *J* = 3.0 Hz, 1H), 7.46 (td, *J* = 7.3, 1.6 Hz, 1H), 7.43 (td, *J* = 7.5, 1.4 Hz, 1H), 7.36 (dd, *J* = 8.9, 3.0 Hz, 1H), 7.28 (d, *J* = 7.3 Hz, 1H), 4.43 (t, *J* = 6.5 Hz, 2H), 3.94 (s, 3H), 3.10 (t, *J* = 6.5 Hz, 2H) ppm; ^{13}C NMR (150

MHz, $CDCl_3$) δ 161.5, 158.3, 147.4, 142.5, 136.7, 131.3, 129.7, 129.3, 127.7, 127.6, 127.5, 124.6, 121.5, 106.2, 55.8, 39.8, 27.5 ppm; HRMS (ESI) calcd for $[C_{17}H_{14}N_2O_2+H]^+$ 279.1128, found 279.1125.

10-(trifluoromethoxy)-5H-isoquinolino[1,2-b]quinazolin-8(6H)-one (3g)

Yield 88%; white solid, m.p. 194-196 ^oC; ^1H NMR (600 MHz, $CDCl_3$) δ 8.47 (dd, *J* = 7.8, 1.4 Hz, 1H), 8.14 (dd, *J* = 2.7, 1.3 Hz, 1H), 7.81 (d, *J* = 8.9 Hz, 1H), 7.58 (dd, *J* = 8.9, 2.7 Hz, 1H), 7.50 (td, *J* = 7.4, 1.5 Hz, 1H), 7.45 (td, *J* = 7.6, 1.4 Hz, 1H), 7.30 (d, *J* = 7.4 Hz, 1H), 4.42 (t, *J* = 6.5 Hz, 2H), 3.12 (t, *J* = 6.5 Hz, 2H) ppm; ^{13}C NMR (150 MHz, $CDCl_3$) δ 160.9, 149.8, 147.1, 147.0, 146.3, 137.0, 132.0, 129.7, 129.2, 128.1, 127.7, 127.6, 127.6, 121.6, 121.3, 119.6, 118.2, 39.8, 27.4 ppm; HRMS (ESI) calcd for $[C_{17}H_{11}F_3N_2O_2+H]^+$ 333.0845, found 333.0840.

10-chloro-5H-isoquinolino[1,2-b]quinazolin-8(6H)-one (3h)

Yield 80%; white solid, m.p. 179-180 ^oC; ^1H NMR (600 MHz, $CDCl_3$) δ 8.45 (d, *J* = 7.8 Hz, 1H), 8.26 (d, *J* = 2.3 Hz, 1H), 7.73 – 7.63 (m, 2H), 7.48 (dd, *J* = 7.9, 6.5 Hz, 1H), 7.43 (t, *J* = 7.6 Hz, 1H), 7.28 (d, *J* = 7.5 Hz, 1H), 4.40 (t, *J* = 6.5 Hz, 2H), 3.10 (t, *J* = 6.5 Hz, 2H) ppm; ^{13}C NMR (150 MHz, $CDCl_3$) δ 160.7, 149.6, 146.3, 137.0, 134.7, 132.2, 132.0, 129.3, 129.3, 128.0, 127.7, 127.6, 126.2, 121.7, 39.8, 27.4 ppm; HRMS (ESI) calcd for $[C_{16}H_{11}ClN_2O+H]^+$ 283.0633, found 283.0631.

10-bromo-5H-isoquinolino[1,2-b]quinazolin-8(6H)-one (3i)

Yield 77%; light yellow solid, m.p. 190-192 ^{o}C; ^{1}H NMR (600 MHz, $CDCl_3$) δ 8.43 (dd, *J* = 7.8, 1.4 Hz, 1H), 8.12 (d, *J* = 8.5 Hz, 1H), 7.92 (d, *J* = 1.9 Hz, 1H), 7.52 (dd, *J* = 8.5, 1.9 Hz, 1H), 7.47 (td, *J* = 7.4, 1.5 Hz, 1H), 7.45 – 7.39 (m, 1H), 7.27 (d, *J* = 7.5 Hz, 1H), 4.37 (t, *J* = 6.5 Hz, 2H), 3.09 (t, *J* = 6.5 Hz, 2H) ppm; ^{13}C NMR (150 MHz, $CDCl_3$) δ 160.6, 149.8, 146.6, 137.4, 137.0, 132.0, 129.4, 129.4, 129.3, 128.1, 127.7, 127.6, 122.1, 119.9, 39.8, 27.4 ppm; HRMS (ESI) calcd for $[C_{16}H_{11}ClN_2O+H]^+$ 327.0126, found 327.0126.

10-iodo-5H-isoquinolino[1,2-b]quinazolin-8(6H)-one (3j)

Yield 75%; white solid, m.p. 194-196 ^{o}C; ^{1}H NMR (600 MHz, $CDCl_3$) δ 8.62 (d, *J* = 2.1 Hz, 1H), 8.44 (dd, *J* = 7.9, 1.4 Hz, 1H), 7.98 (dd, *J* = 8.6, 2.1 Hz, 1H), 7.47 (d, *J* = 8.3 Hz, 2H), 7.46 – 7.38 (m, 1H), 7.28 (d, *J* = 7.5 Hz, 1H), 4.39 (t, *J* = 6.5 Hz, 2H), 3.09 (t, *J* = 6.5 Hz, 2H) ppm; ^{13}C NMR (150 MHz, $CDCl_3$) δ 160.3, 149.9, 147.1, 142.9, 137.0, 135.7, 132.0, 129.4, 129.3, 128.1, 127.7, 127.6, 122.3, 90.8, 39.8, 27.4 ppm; HRMS (ESI) calcd for $[C_{16}H_{11}IN_2O+H]^+$ 374.9989, found 374.9986.

9-chloro-5H-isoquinolino[1,2-b]quinazolin-8(6H)-one (3k)

Yield 79%; light yellow solid, m.p. 200-202 ^{o}C; ^{1}H NMR (600 MHz, $CDCl_3$) δ 8.45 (dd, *J* = 7.8, 1.4 Hz, 1H), 7.67 (dd, *J* = 8.2, 1.2 Hz, 1H), 7.58 (t, *J* = 8.0 Hz, 1H), 7.49 (td, *J* = 7.4, 1.4 Hz, 1H), 7.46 – 7.41 (m, 2H), 7.29 (d, *J* = 7.5 Hz, 1H), 4.37 (t, *J* = 6.5 Hz, 2H), 3.10 (t, *J* = 6.5 Hz, 2H) ppm; ^{13}C NMR (150 MHz, $CDCl_3$) δ: 159.8, 150.2, 150.0, 137.2,

134.1, 133.5, 132.0, 129.2, 129.0, 128.0, 127.6, 127.5, 127.0, 117.9, 39.6, 27.4; HRMS (ESI) calcd for $[C_{16}H_{11}ClN_2O+H]+$ 283.0633, found 283.0631.

9-bromo-5H-isoquinolino[1,2-b]quinazolin-8(6H)-one (3l)

Yield 81%; white solid, m.p. 220-222 ^{o}C; ^{1}H NMR (600 MHz, $CDCl_3$) δ 8.44 (dd, *J* = 7.8, 1.4 Hz, 1H), 7.70 (dd, *J* = 8.1, 1.2 Hz, 1H), 7.68 (dd, *J* = 7.8, 1.2 Hz, 1H), 7.50 (d, *J* = 7.8 Hz, 1H), 7.47 (dd, *J* = 7.0, 1.7 Hz, 1H), 7.45 – 7.40 (m, 1H), 7.28 (d, *J* = 7.4 Hz, 1H), 4.36 (t, *J* = 6.5 Hz, 2H), 3.10 (t, *J* = 6.5 Hz, 2H) ppm; ^{13}C NMR (150 MHz, $CDCl_3$) δ 159.9, 150.1, 149.7, 137.2, 133.8, 133.0, 132.0, 129.0, 128.0, 127.7, 127.6, 127.5, 121.5, 118.8, 39.7, 27.4 ppm; HRMS (ESI) calcd for $[C_{16}H_{11}BrN_2O+H]^+$ 327.0128, found 327.0127.

9,11-dichloro-5H-isoquinolino[1,2-b]quinazolin-8(6H)-one (3m)

Yield 69%; Light yellow solid, m.p. 230-232 ^{o}C; ^{1}H NMR (600 MHz, $CDCl_3$) δ 8.42 (dd, *J* = 7.9, 1.4 Hz, 1H), 7.66 (d, *J* = 2.1 Hz, 1H), 7.51 (td, *J* = 7.5, 1.4 Hz, 1H), 7.45 (dd, *J* = 7.8, 1.3 Hz, 1H), 7.42 (d, *J* = 2.2 Hz, 1H), 7.29 (d, *J* = 7.5 Hz, 1H), 4.35 (t, *J* = 6.5 Hz, 2H), 3.10 (t, *J* = 6.5 Hz, 2H) ppm; ^{13}C NMR (150 MHz, $CDCl_3$) δ 163.4, 159.3, 151.0, 150.7, 139.3, 137.3, 135.3, 132.4, 129.0, 128.7, 128.1, 127.7, 127.6, 126.4, 116.4, 39.6, 27.3 ppm; HRMS (ESI) calcd for $[C_{16}H_{10}Cl_2N_2O+H]^+$ 317.0243, found 317.0240.

2-methyl-5H-isoquinolino[1,2-b]quinazolin-8(6H)-one (3n)

Yield 86%; white solid, m.p. 165-167 °C; ^{1}H NMR (600 MHz, $CDCl_3$) δ 8.31 (d, *J* = 8.0 Hz, 1H), 8.29 (s, 1H), 7.80 – 7.72 (m, 2H), 7.45 (t, *J* = 7.4 Hz, 1H), 7.28 (d, *J* = 7.7 Hz, 1H), 7.17 (d, *J* = 7.7 Hz, 1H), 4.39 (t, *J* = 6.5 Hz, 2H), 3.05 (t, *J* = 6.5 Hz, 2H), 2.45 (s, 3H) ppm; ^{13}C NMR (150 MHz, $CDCl_3$) δ 161.7, 149.6, 147.8, 137.4, 134.2, 132.6, 129.3, 128.2, 127.5, 127.4, 126.9, 126.4, 120.8, 39.8, 27.1, 21.2 ppm; HRMS (ESI) calcd for $[C_{17}H_{14}N_2O+H]^+$ 263.1179, found 263.1175.

2-methoxy-5H-isoquinolino[1,2-b]quinazolin-8(6H)-one (3o)

Yield 88%; light yellow solid, m.p. 221-223 °C; ^{1}H NMR (600 MHz, $CDCl_3$) δ 8.45 (d, *J* = 8.7 Hz, 1H), 8.31 (dt, *J* = 8.0, 1.1 Hz, 1H), 7.77 – 7.72 (m, 2H), 7.44 (dt, *J* = 8.1, 4.3 Hz, 1H), 6.98 (dd, *J* = 8.8, 2.6 Hz, 1H), 6.79 (d, *J* = 2.5 Hz, 1H), 4.46 – 4.39 (m, 2H), 3.91 (s, 3H), 3.09 (t, *J* = 6.5 Hz, 2H) ppm; ^{13}C NMR (150 MHz, $CDCl_3$) δ 162.5, 161.8, 149.4, 148.1, 139.0, 134.1, 130.1, 127.3, 126.8, 126.0, 122.2, 120.5, 113.7, 112.2, 55.5, 39.57, 27.8 ppm; HRMS (ESI) calcd for $[C_{17}H_{14}N_2O_2+H]^+$ 279.1128, found 279.1125.

2-fluoro-5H-isoquinolino[1,2-b]quinazolin-8(6H)-one (3p)

Yield 75%; light yellow solid, m.p. 211-213 °C; ^{1}H NMR (600 MHz, $CDCl_3$) δ 8.31 (d, *J* = 7.9 Hz, 1H), 8.19 (dd, *J* = 9.6, 2.7 Hz, 1H), 7.82 – 7.74 (m, 2H), 7.48 (ddd, *J* = 8.1, 5.1, 3.1 Hz, 1H), 7.30 – 7.24 (m, 1H), 7.18 (td, *J* = 8.2, 2.7 Hz, 1H), 4.41 (t, *J* = 6.5 Hz, 2H), 3.08 (t, *J* = 6.5 Hz, 2H) ppm; ^{13}C NMR (150 MHz, $CDCl_3$) δ 163.0, 161.6, 161.3, 148.4,

148.3, 147.6, 134.4, 132.7, 132.7, 131.4, 131.4, 129.2, 129.2, 127.7, 126.9, 126.9, 120.9, 119.0, 118.8, 114.6, 114.5, 39.7, 26.8 ppm; HRMS (ESI) calcd for $[C_{16}H_{11}FN_2O+H]^+$ 267.0928, found 267.0924.

2-nitro-5H-isoquinolino[1,2-b]quinazolin-8(6H)-one (3q)

Yield 66%; light yellow solid, m.p. 289-290 °C; ^{1}H NMR (600 MHz, $CDCl_3$) δ 9.38 (d, J = 2.4 Hz, 1H), 8.35 (ddd, J = 8.2, 4.8, 1.9 Hz, 2H), 7.90 – 7.79 (m, 2H), 7.55 (ddd, J = 8.1, 6.9, 1.4 Hz, 1H), 7.51 (d, J = 8.3 Hz, 1H), 4.49 (t, J = 6.5 Hz, 2H), 3.26 (t, J = 6.5 Hz, 2H) ppm; ^{13}C NMR (150 MHz, $CDCl_3$) δ 161.2, 147.8, 147.3, 147.2, 143.4, 134.6, 131.2, 128.8, 127.9, 127.4, 126.9, 125.8, 123.4, 120.9, 100.0, 39.0, 27.6 ppm; HRMS (ESI) calcd for $[C_{16}H_{11}N_3O_2+H]^+$ 294.0812, found 294.0813.

2,3-dimethoxy-5H-isoquinolino[1,2-b]quinazolin-8(6H)-one (3r)

Yield 89%; Light yellow solid, m.p. 207-209 °C; δ ^{1}H NMR (600 MHz, $CDCl_3$) δ 8.29 (d, J = 7.9 Hz, 1H), 7.98 (s, 1H), 7.79 – 7.70 (m, 2H), 7.43 (t, J = 7.2 Hz, 1H), 6.73 (s, 1H), 4.40 (t, J = 6.5 Hz, 2H), 4.04 (s, 3H), 3.96 (s, 3H), 3.03 (t, J = 6.5 Hz, 2H) ppm; ^{13}C NMR (150 MHz, $CDCl_3$) δ 161.8, 152.2, 149.3, 148.6, 147.9, 134.1, 130.9, 127.3, 126.9, 126.1, 121.8, 120.5, 110.1, 109.7, 56.2, 56.1, 39.7, 27.0 ppm; HRMS (ESI) calcd for $[C_{18}H_{16}N_2O_3+H]^+$ 309.1234, found 309.1228.

***isoindolo[1,2-b]quinazolin-10(12H)-one* (3s)**

Yield 81%; White yellow solid; ^{1}H NMR (600 MHz, $CDCl_3$) δ 8.38 (dd, J = 8.0, 1.5 Hz, 1H), 8.18 (d, J = 7.7 Hz, 1H), 7.86 – 7.81 (m, 1H),

7.79 (ddd, *J* = 8.3, 6.9, 1.5 Hz, 1H), 7.64 (d, *J* = 6.2 Hz, 2H), 7.58 (td, *J* = 6.7, 5.6, 2.6 Hz, 1H), 7.50 (ddd, *J* = 8.1, 6.9, 1.3 Hz, 1H), 5.16 (s, 2H) ppm; ^{13}C NMR (150 MHz, $CDCl_3$) δ 160.6, 154.9, 149.5, 139.7, 134.2, 132.7, 132.3, 128.9, 127.4, 126.5, 126.4, 123.5, 120.6, 49.8 ppm. HRMS (ESI) calcd for $[C_{15}H_{10}N_2O+H]^+$ 235.0865, found 235.0864.

8,8a,13,13a-tetrahydroindolo[2',3':3,4]pyrido[2,1-b]quinazolin-5(7H)-one (Rutaecarpine) (3t)

Yield 80%; Light yellow solid, m.p. 260-261 °C; ^{1}H NMR (600 MHz, DMSO-d_6) δ 11.85 (s, 1H), 8.15 (dd, *J* = 8.0, 1.6 Hz, 1H), 7.83 – 7.73 (m, 1H), 7.67 (d, *J* = 8.1 Hz, 1H), 7.61 (d, *J* = 7.9 Hz, 1H), 7.50 (d, *J* = 8.3 Hz, 1H), 7.44 (t, *J* = 7.5 Hz, 1H), 7.26 (t, *J* = 7.6 Hz, 1H), 7.07 (t, *J* = 7.5 Hz, 1H), 4.43 (t, *J* = 6.8 Hz, 2H), 3.15 (t, *J* = 6.8 Hz, 2H) ppm; ^{13}C NMR (150 MHz, DMSO-d_6) δ 161.0, 147.8, 145.7, 139.1, 134.7, 127.5, 127.0, 126.8, 126.3, 125.4, 125.1, 121.2, 120.3, 120.1, 118.2, 113.0, 41.2, 19.4 ppm.

3. Cyclic Voltammetry Experiments

Cyclic voltammetry experiments have been conducted to determine the redox potential of the starting material as shown in Figure 2. According to the CV experiment, the oxidation peak potential of TBAI are 1.75 V vs Ag/AgCl, which is lower than that of **2a** (2.2 V vs Ag/AgCl).

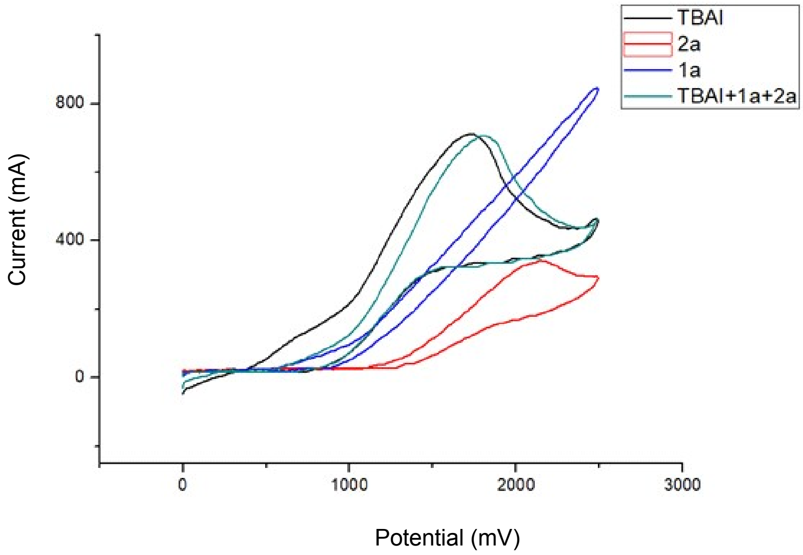

Figure 2. Cyclic voltammograms of related compounds in 0.1 M $NH_4{}^tBu_4BF_4$/EtOH using glass carbon as the working electrode, Pt wire and Ag/AgCl (0.1 M in CH_3CN) as the counter and reference electrode at a scan rate of 100 mV/s: (1) TBAI; (2) **2a**; (3) **1a**; (4) TBAI + **1a** + **2a**

4. ^{1}H NMR and ^{13}C NMR of the Products

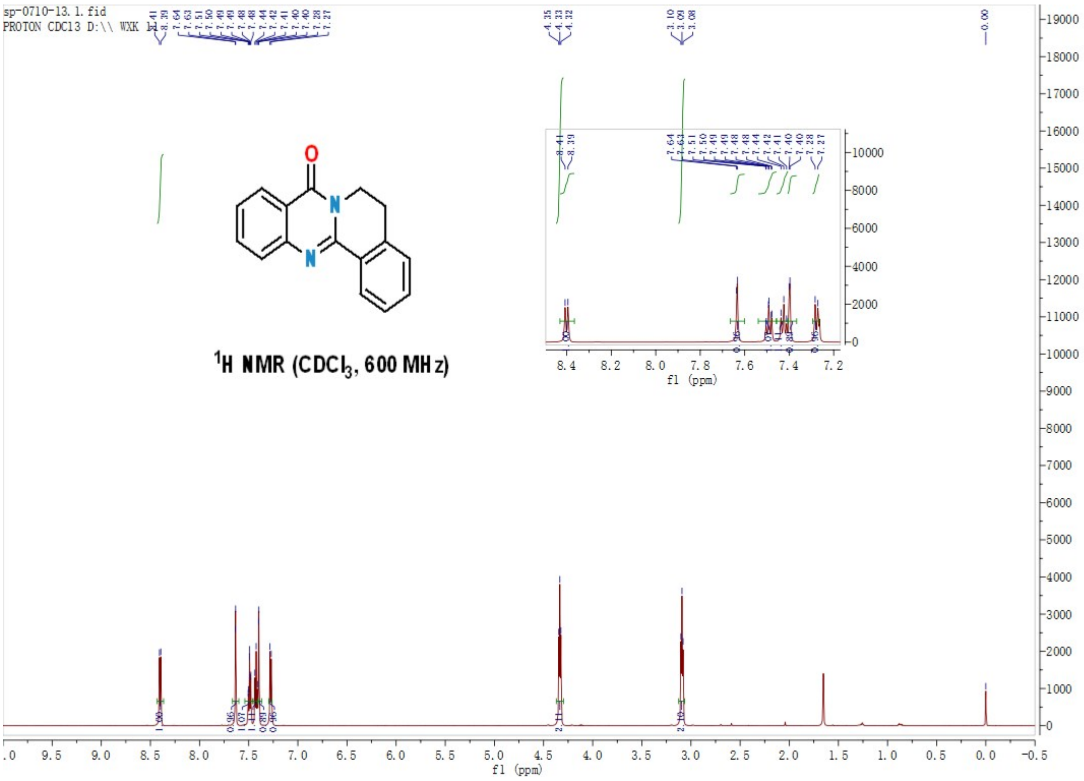

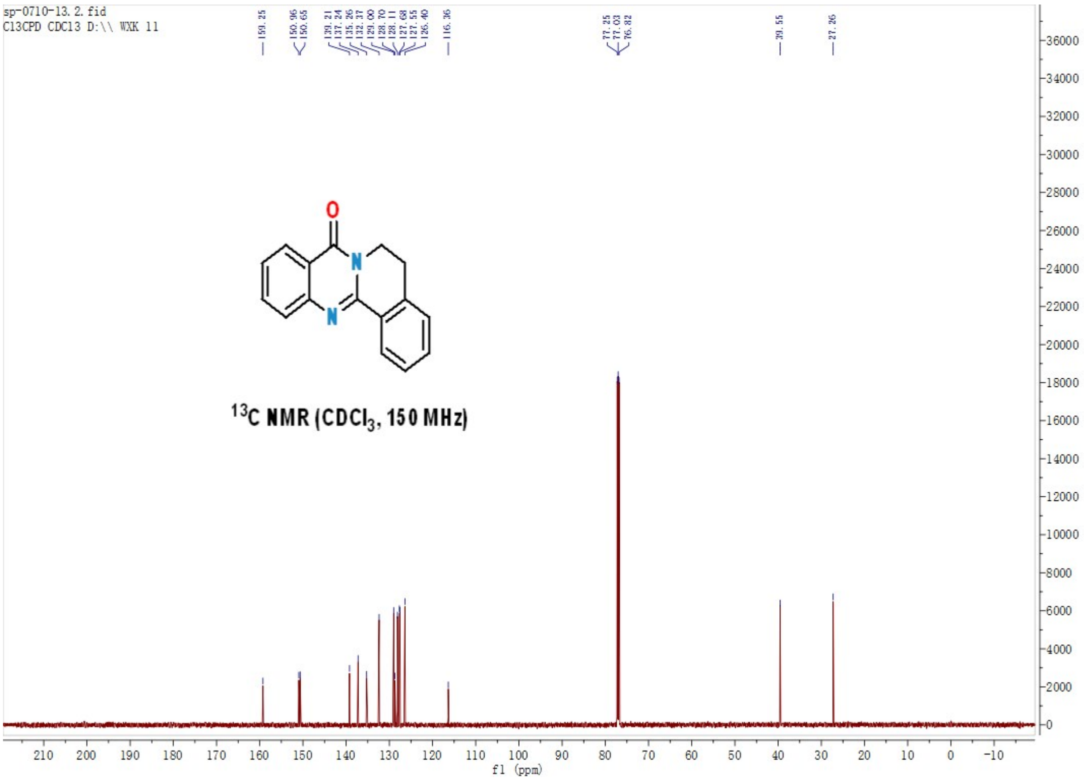

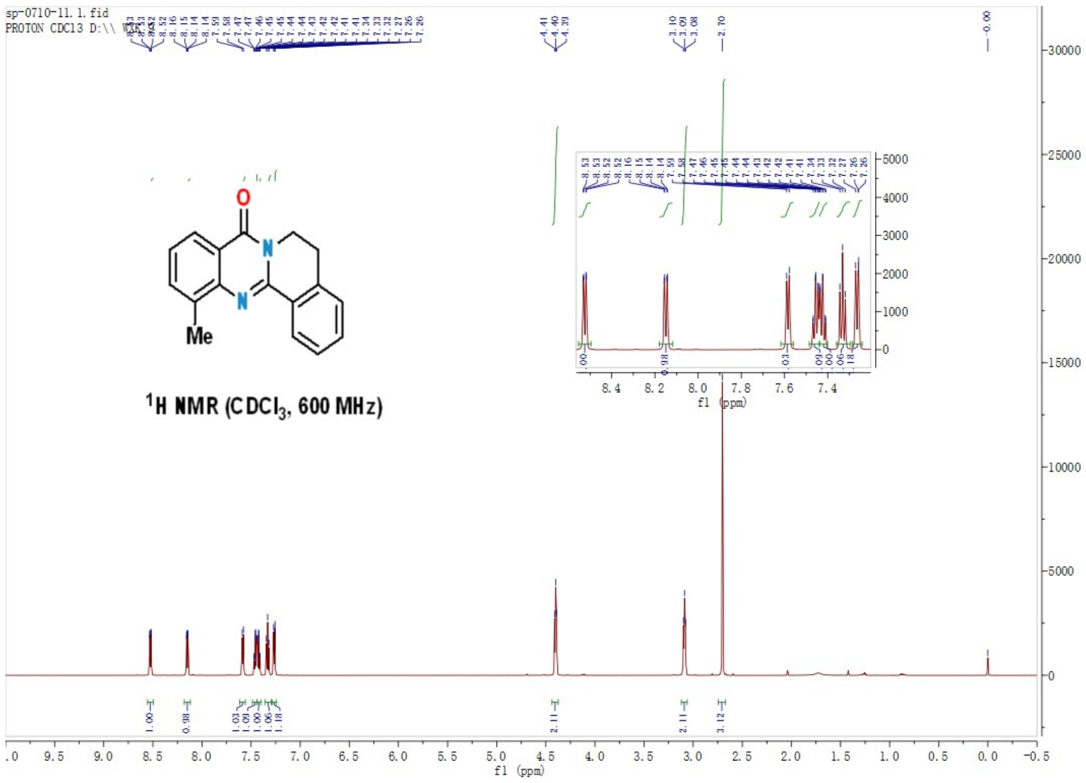
sp-0710-11.1.fid
PROTON CDCl3 D:\\ WXK
¹H NMR (CDCl3, 600 MHz)
Me
f1 (ppm)

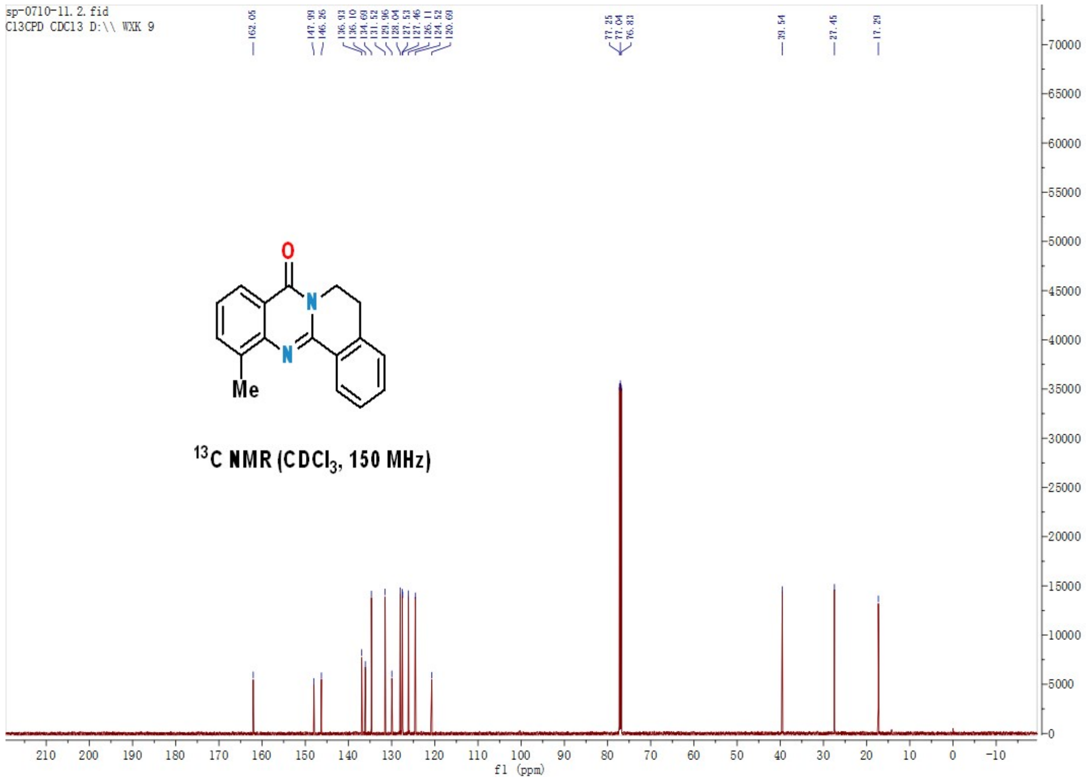
sp-0710-11.2.fid
C13CPD CDCl3 D:\\ WXK 9
162.05
147.99
146.26
136.93
136.10
134.69
131.52
129.96
128.04
127.53
127.46
126.11
124.52
120.69
77.25
77.04
76.83
39.54
27.45
17.29
¹³C NMR (CDCl3, 150 MHz)
Me
f1 (ppm)

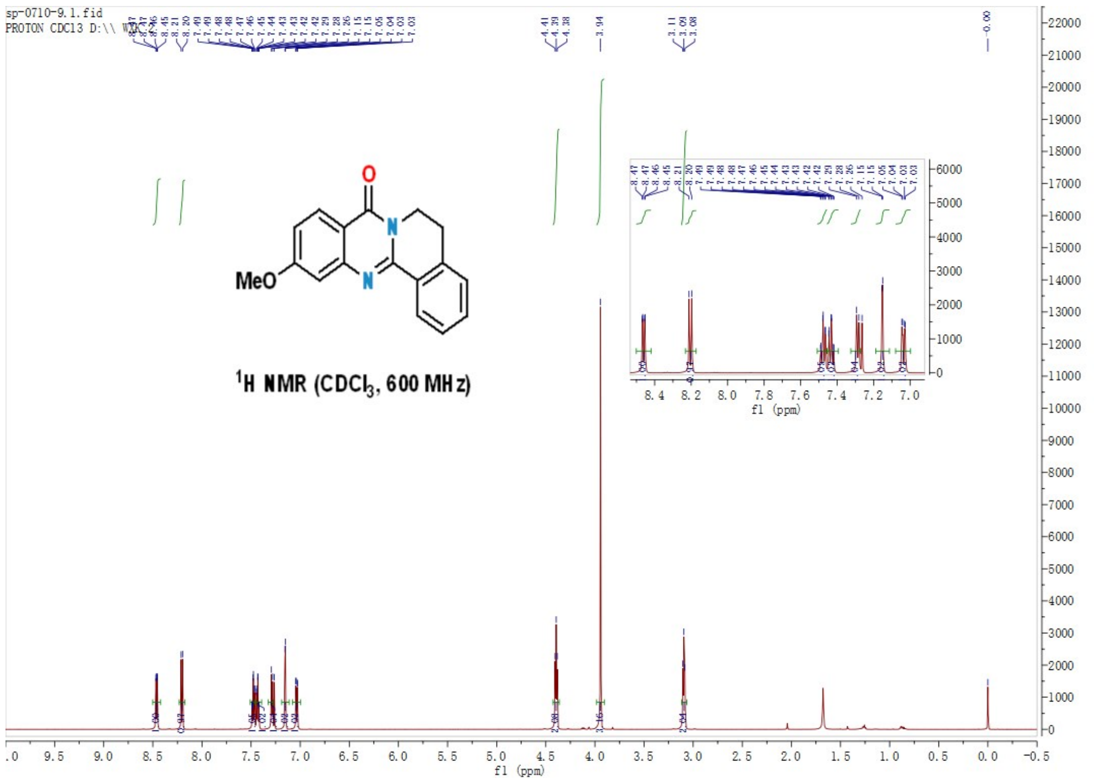

sp-0710-9.1.fid
PROTON CDCl3 D:\\ WXK
4.41
4.39
4.38
3.94
3.11
3.09
3.08
0.00
MeO
O
N
N
1H NMR (CDCl3, 600 MHz)
f1 (ppm)

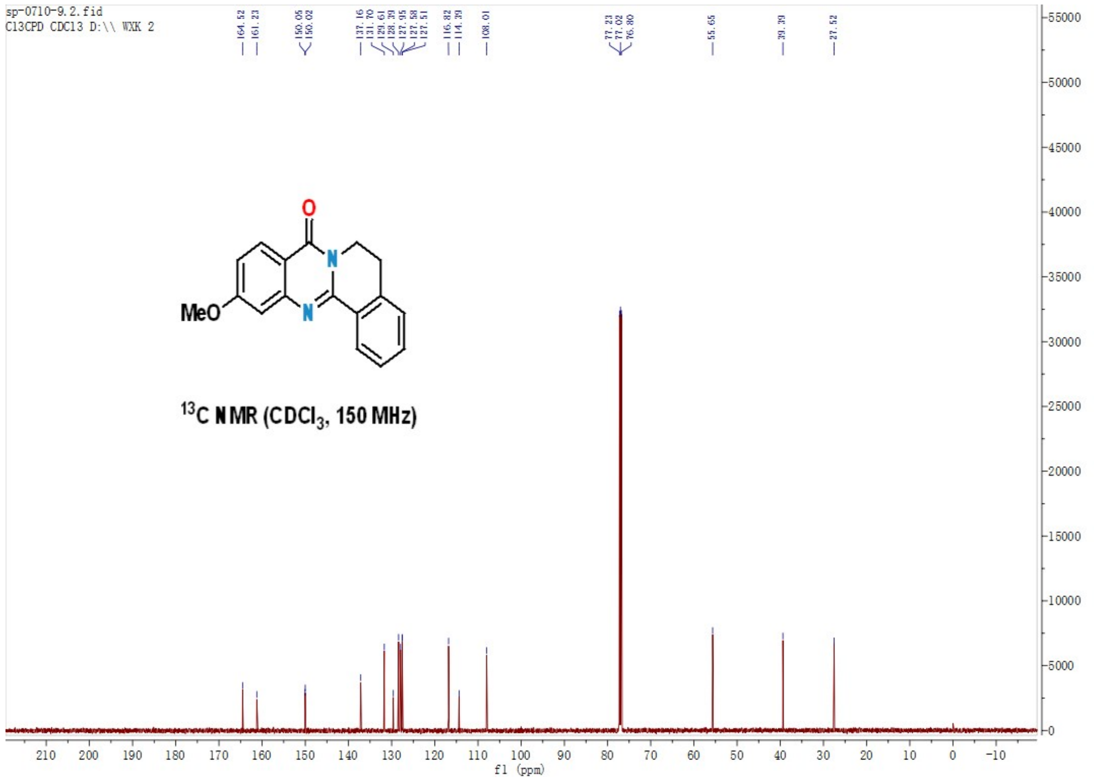

sp-0710-9.2.fid
C13CPD CDCl3 D:\\ WXK 2
164.52
161.23
150.05
150.02
137.16
131.70
129.61
128.39
127.95
127.58
127.51
116.82
114.39
108.01
77.23
77.02
76.80
55.65
39.39
27.52
MeO
O
N
N
13C NMR (CDCl3, 150 MHz)
f1 (ppm)

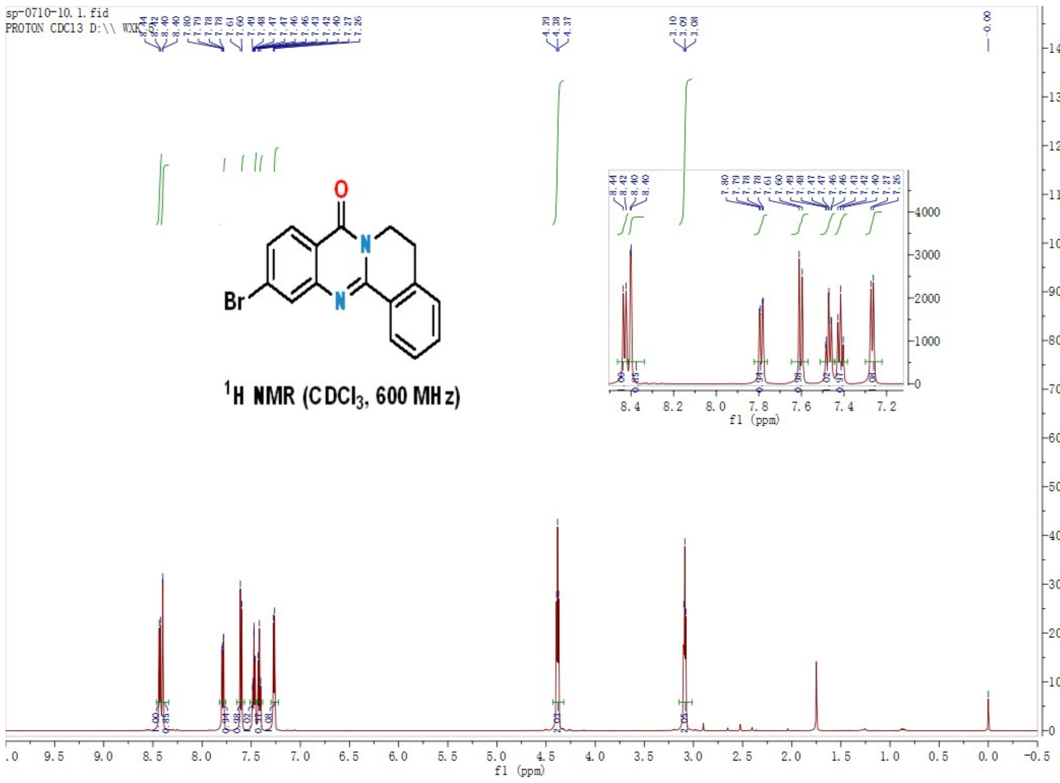

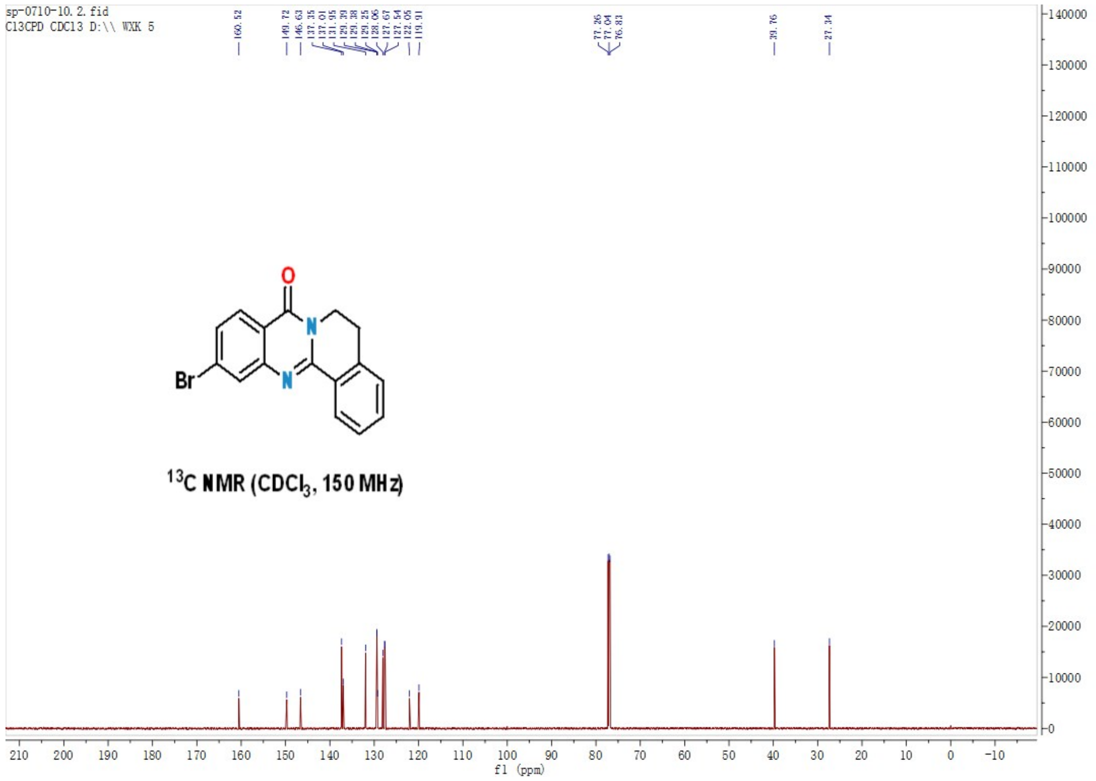

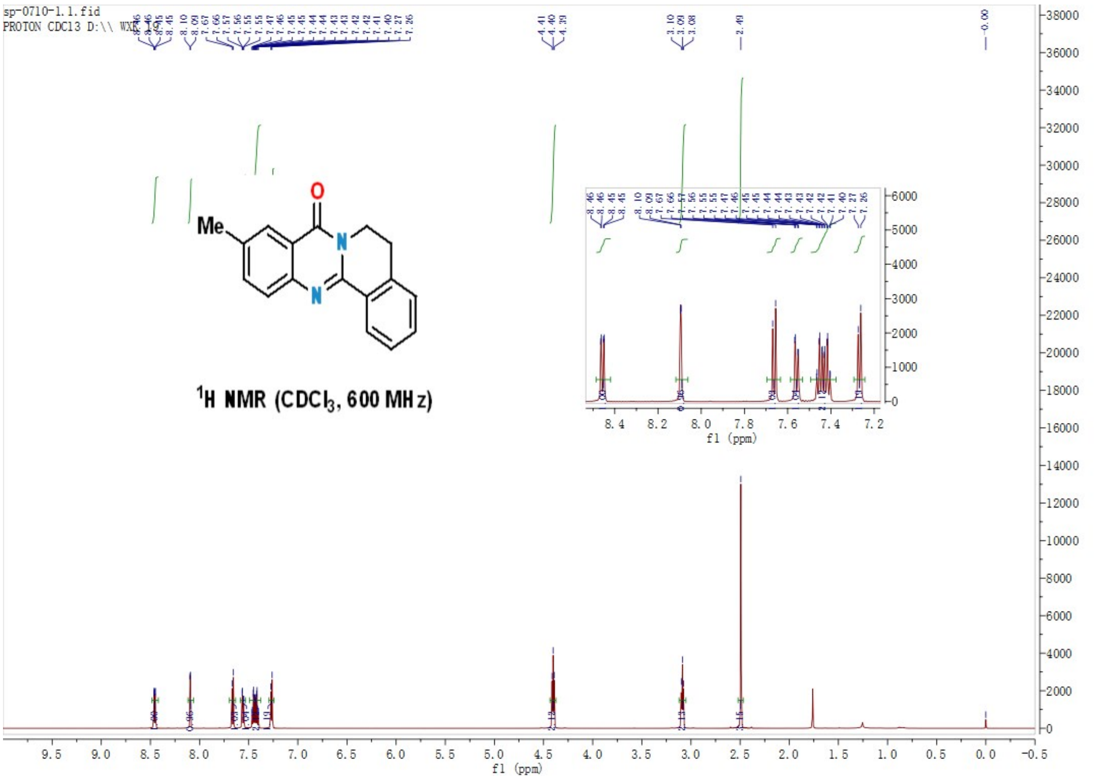
sp-0710-1.1.fid
PROTON CDCl3 D:\\ WXK 19
Me
O
N
N
^{1}H NMR (CDCl$_3$, 600 MHz)
f1 (ppm)

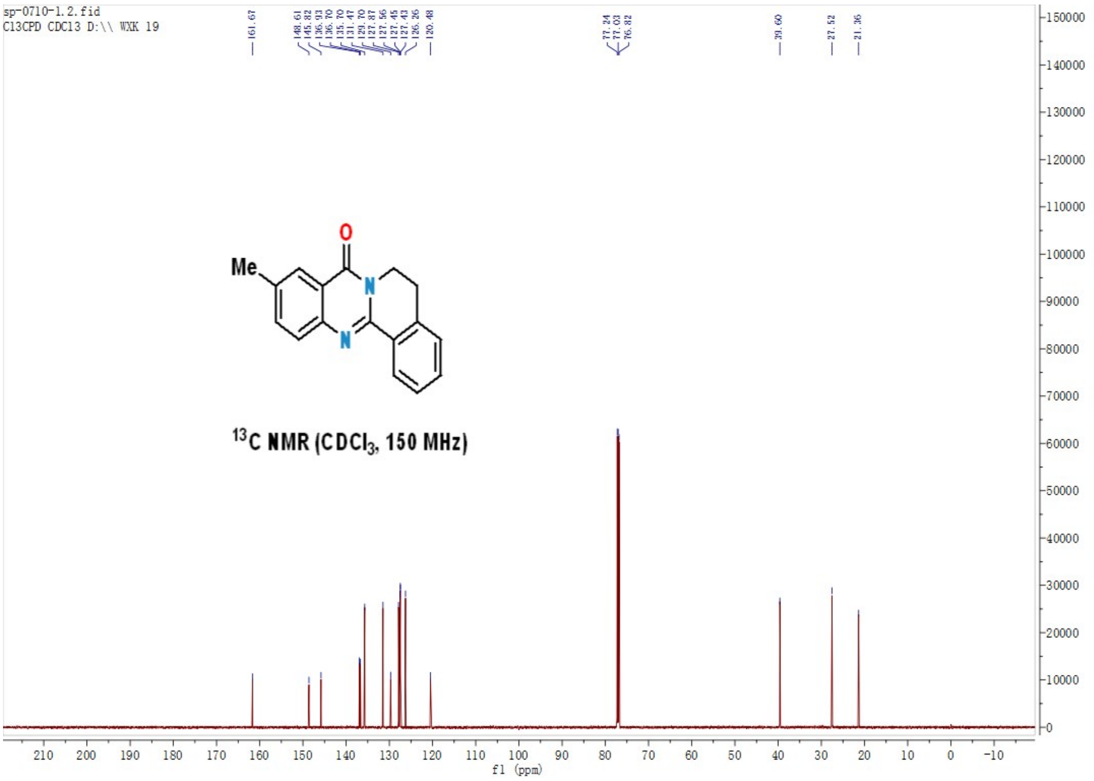
sp-0710-1.2.fid
C13CPD CDCl3 D:\\ WXK 19
161.67
148.61
145.82
136.93
136.70
135.70
131.47
129.70
127.87
127.56
127.45
127.43
126.26
120.48
77.24
77.03
76.82
39.60
27.52
21.36
Me
O
N
N
^{13}C NMR (CDCl$_3$, 150 MHz)
f1 (ppm)

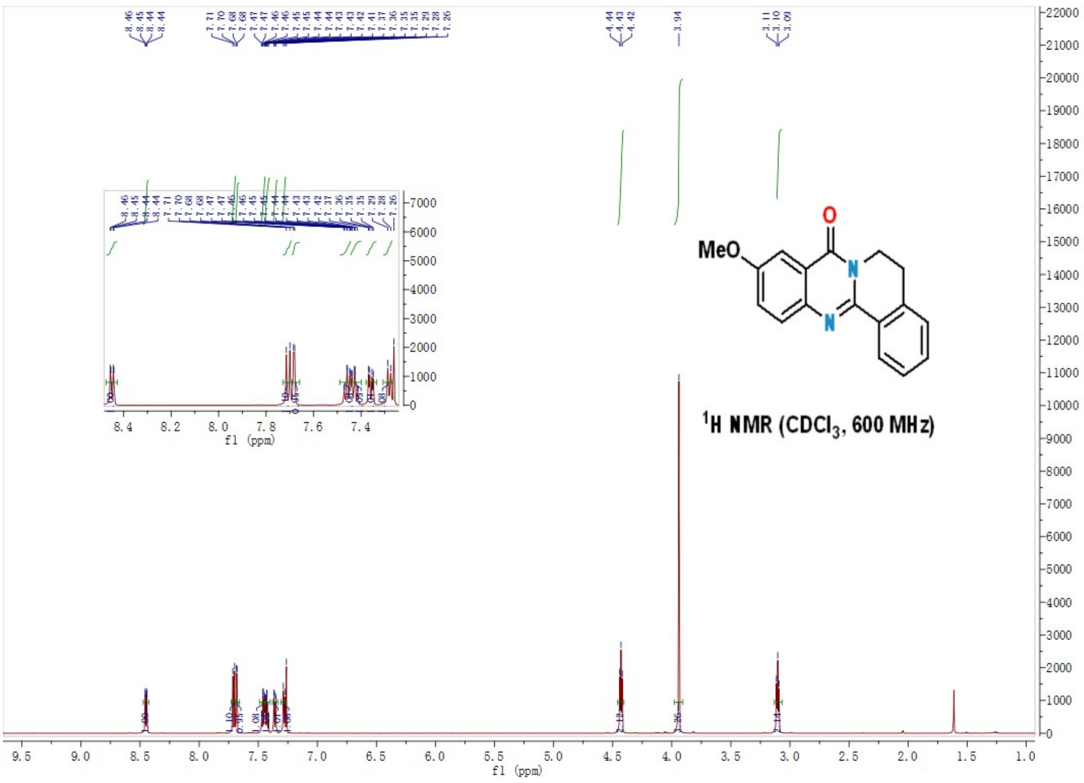

MeO
O
N
N
^{1}H NMR (CDCl$_3$, 600 MHz)
f1 (ppm)

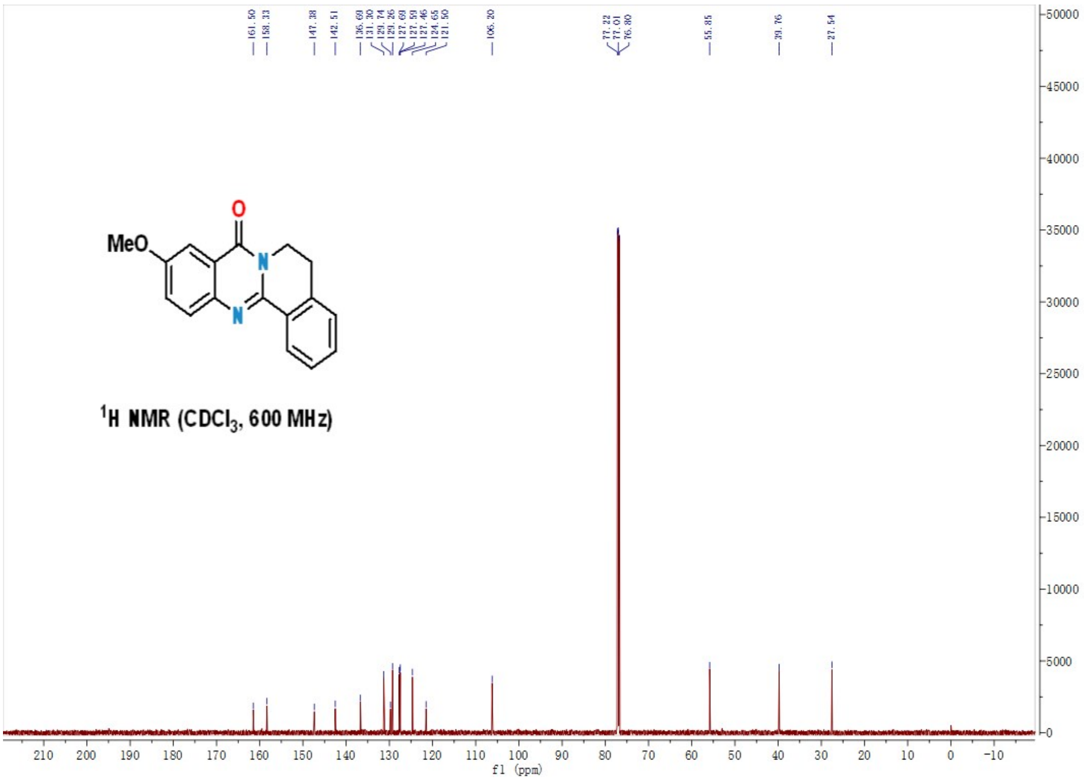

MeO
O
N
N
^{1}H NMR (CDCl$_3$, 600 MHz)
f1 (ppm)

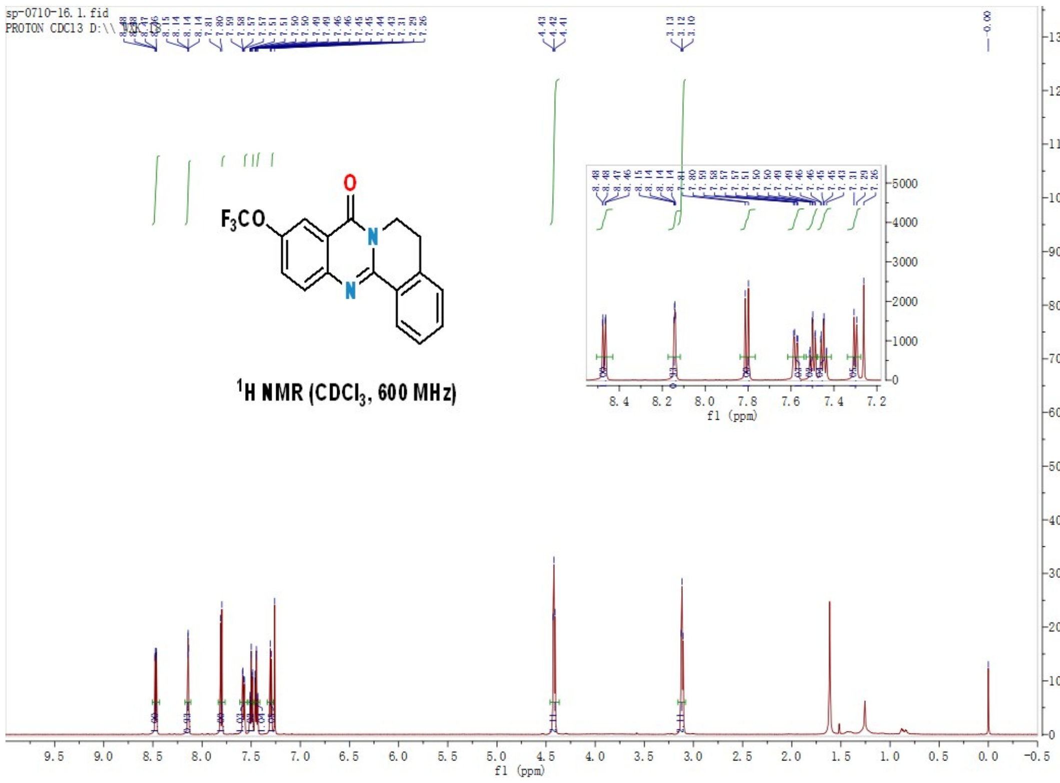
sp-0710-16.1.fid
PROTON CDCl3 D:\\ WXK 18
^{1}H NMR (CDCl$_3$, 600 MHz)
F$_3$CO
f1 (ppm)

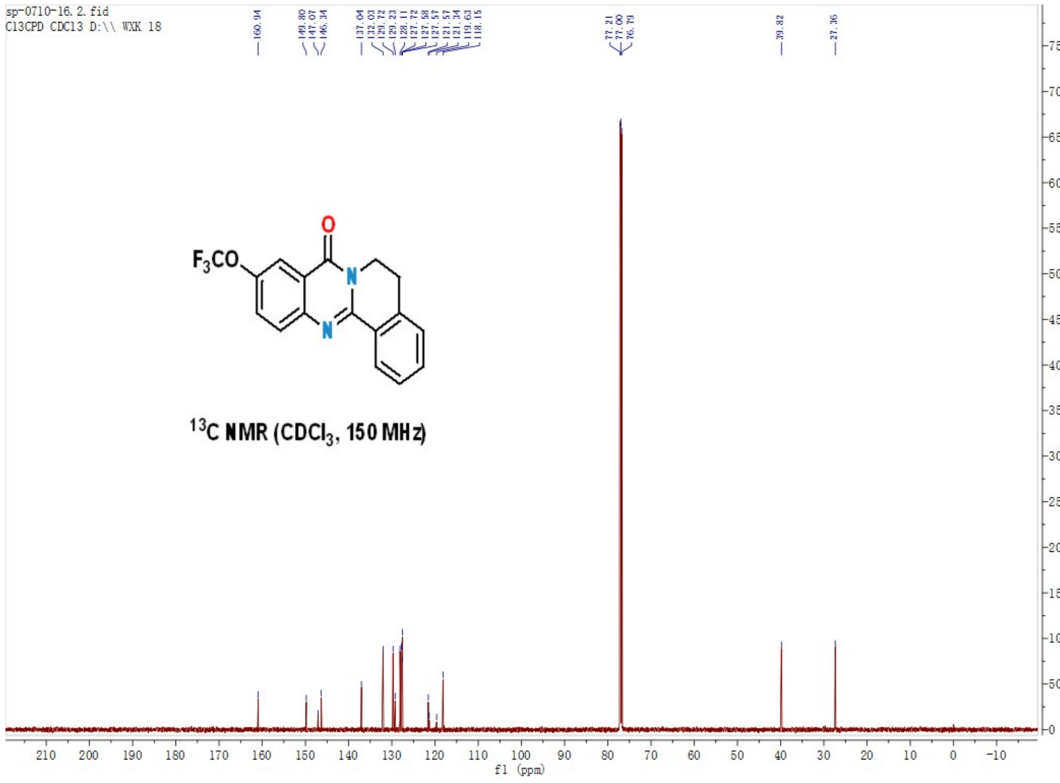
sp-0710-16.2.fid
C13CPD CDCl3 D:\\ WXK 18
160.94
149.80
147.07
146.34
137.04
132.03
129.72
129.23
128.11
127.72
127.58
127.57
121.57
121.34
119.63
118.15
77.21
77.00
76.79
39.82
27.36
^{13}C NMR (CDCl$_3$, 150 MHz)
F$_3$CO
f1 (ppm)

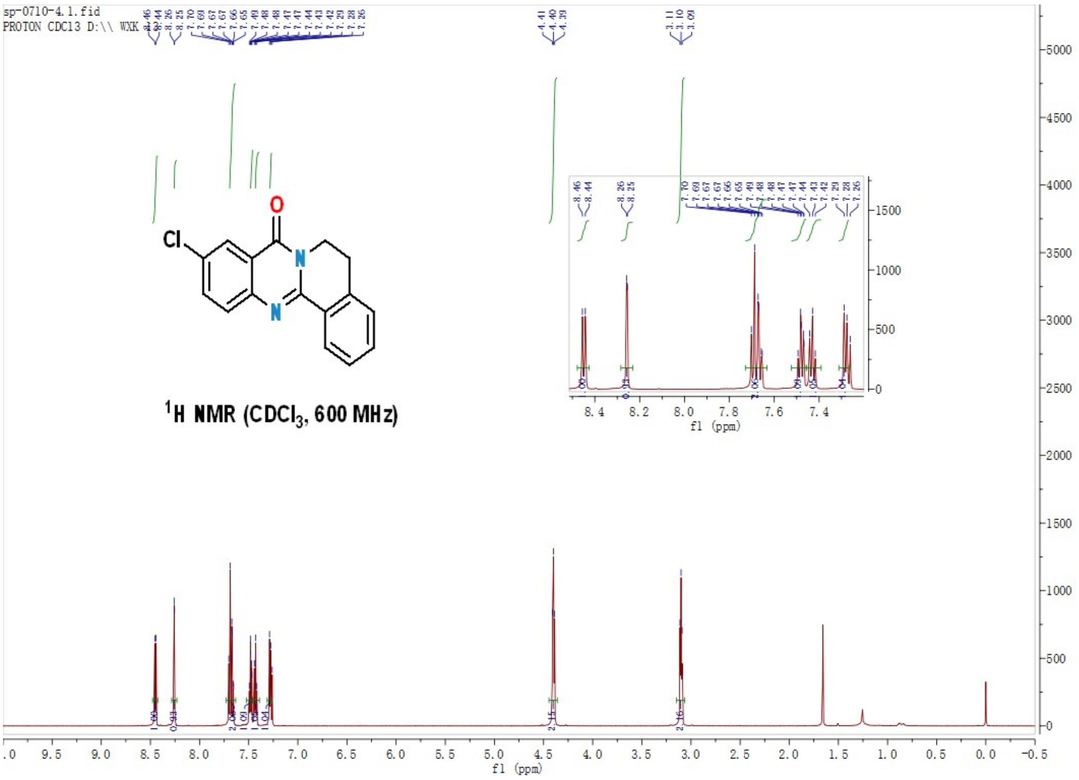
sp-0710-4.1.fid
PROTON CDCl3 D:\\ WXK 13
8.46 8.44 8.26 8.25 7.70 7.69 7.67 7.67 7.66 7.65 7.49 7.48 7.48 7.47 7.44 7.43 7.42 7.29 7.28 7.26
4.41 4.40 4.39
3.11 3.10 3.09
Cl
O
N
N
^{1}H NMR (CDCl$_3$, 600 MHz)
f1 (ppm)

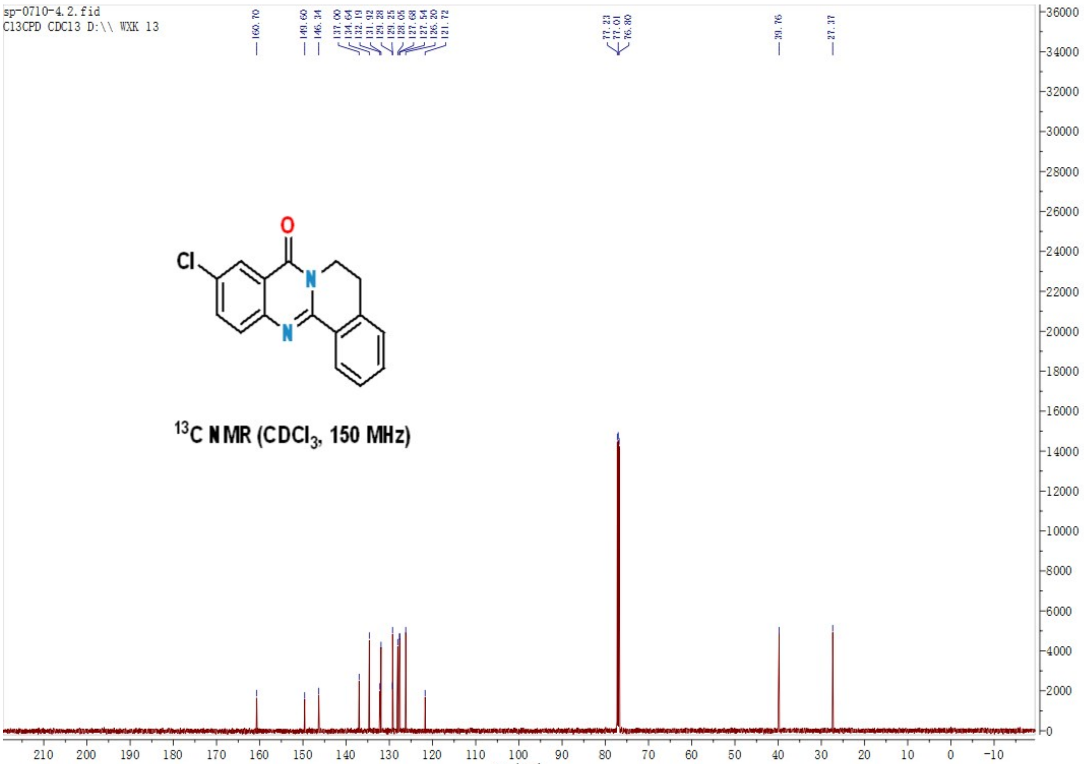
sp-0710-4.2.fid
C13CPD CDCl3 D:\\ WXK 13
160.70
149.60 146.34
137.00 134.64 132.19 131.92 129.28 129.25 128.05 127.68 127.54 126.20 121.72
77.23 77.01 76.80
39.76
27.37
Cl
O
N
N
^{13}C NMR (CDCl$_3$, 150 MHz)
f1 (ppm)

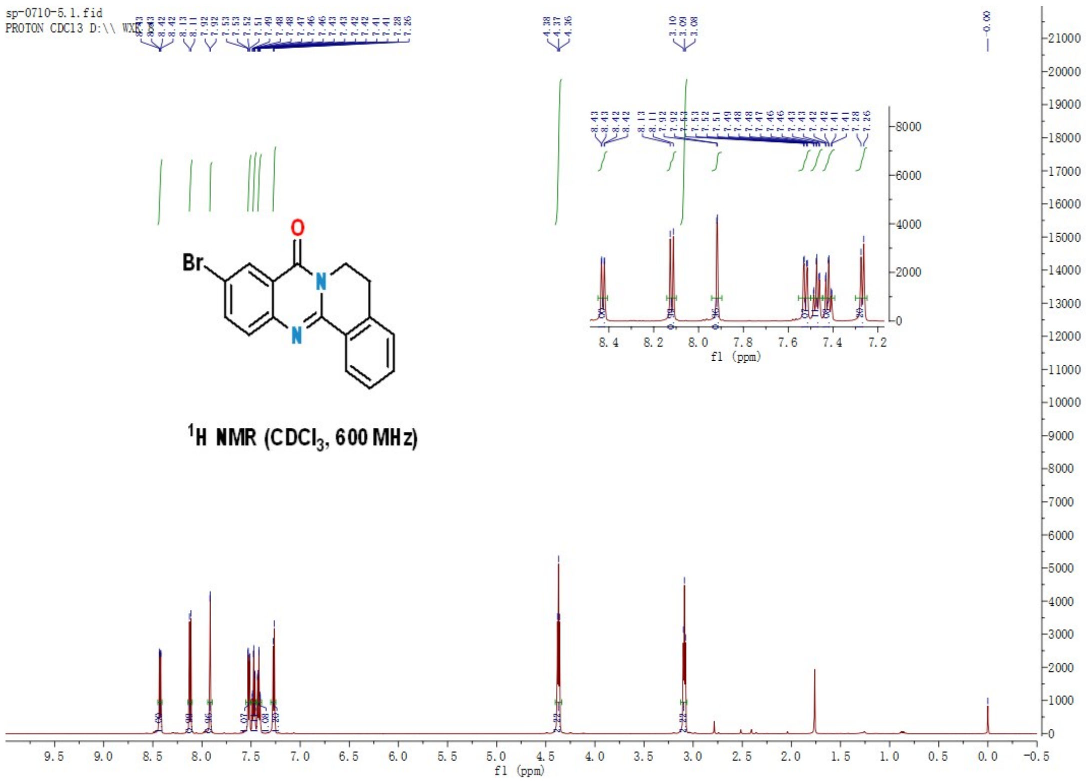

sp-0710-5.1.fid
PROTON CDCl3 D:\\ WXK 8
1H NMR (CDCl3, 600 MHz)
Br
O
N
f1 (ppm)

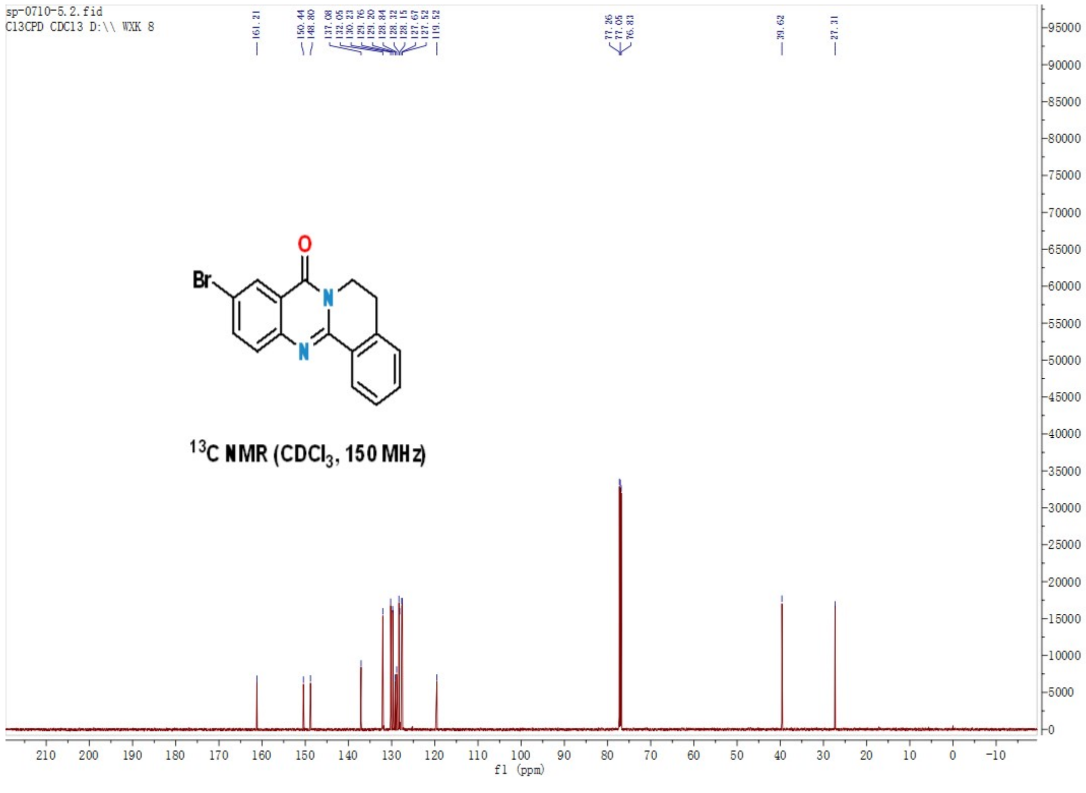

sp-0710-5.2.fid
C13CPD CDCl3 D:\\ WXK 8
161.21
150.44
148.80
137.08
132.05
130.23
129.76
129.20
128.84
128.32
128.15
127.67
127.52
119.52
77.26
77.05
76.83
39.62
27.31
13C NMR (CDCl3, 150 MHz)
Br
O
N
f1 (ppm)

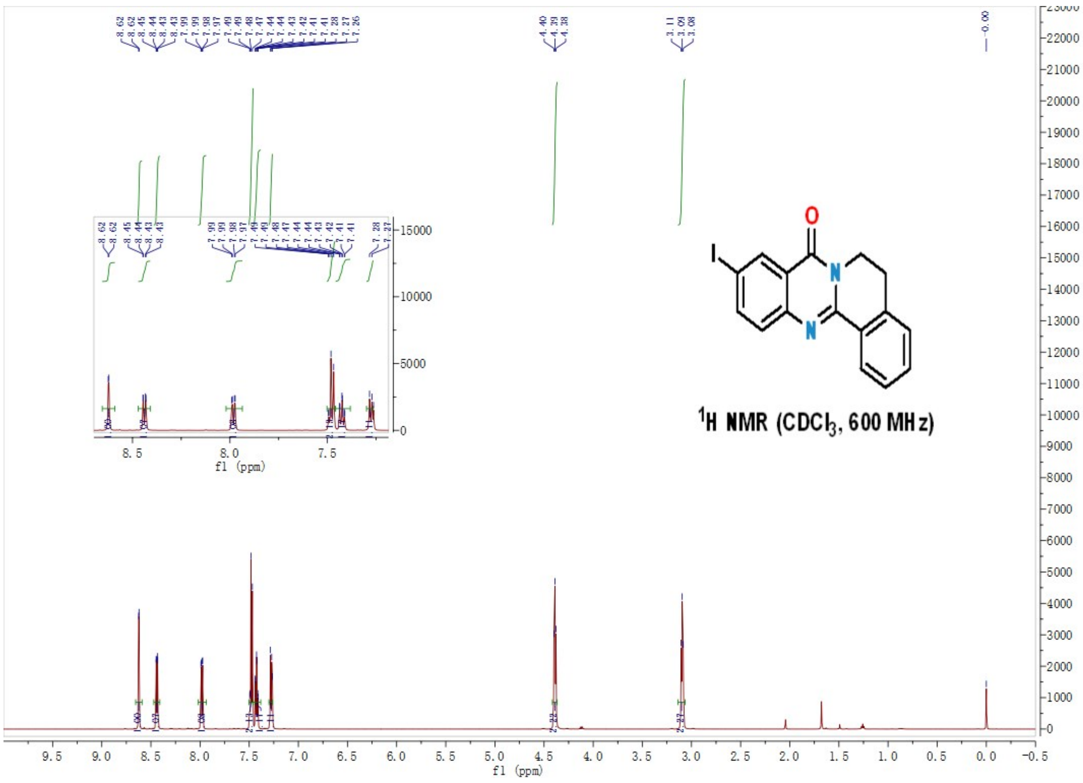
1H NMR (CDCl3, 600 MHz)
f1 (ppm)

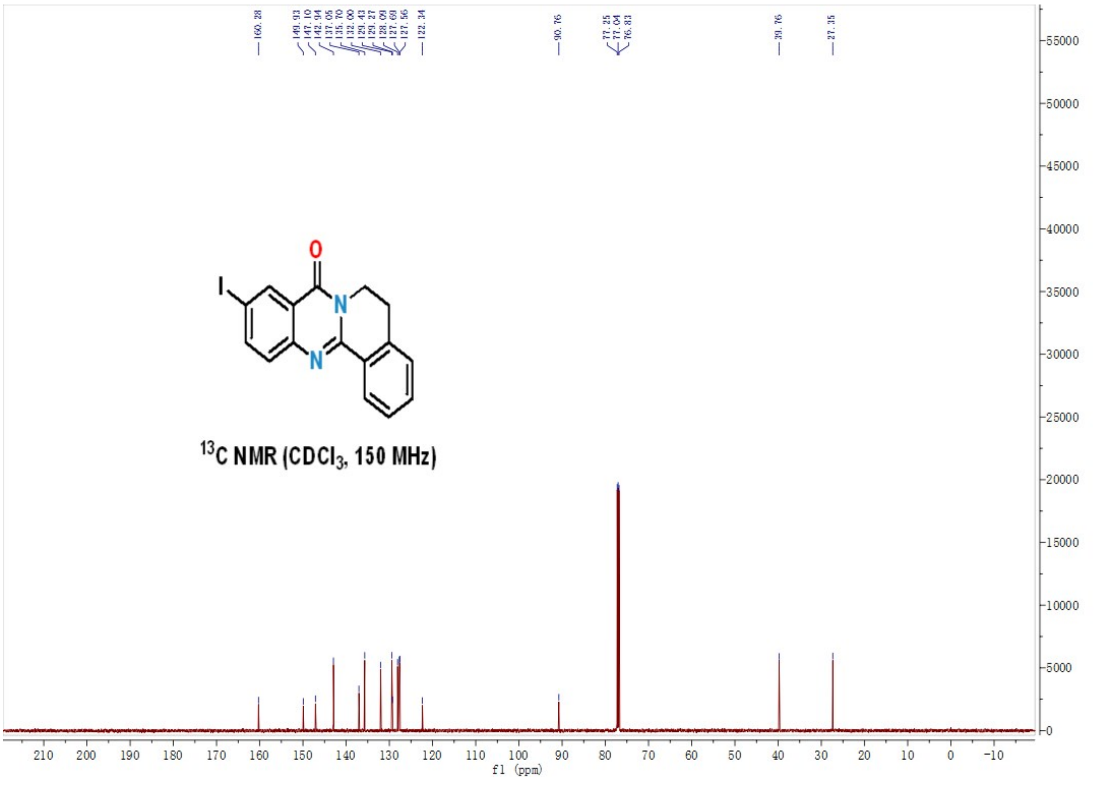
13C NMR (CDCl3, 150 MHz)
f1 (ppm)

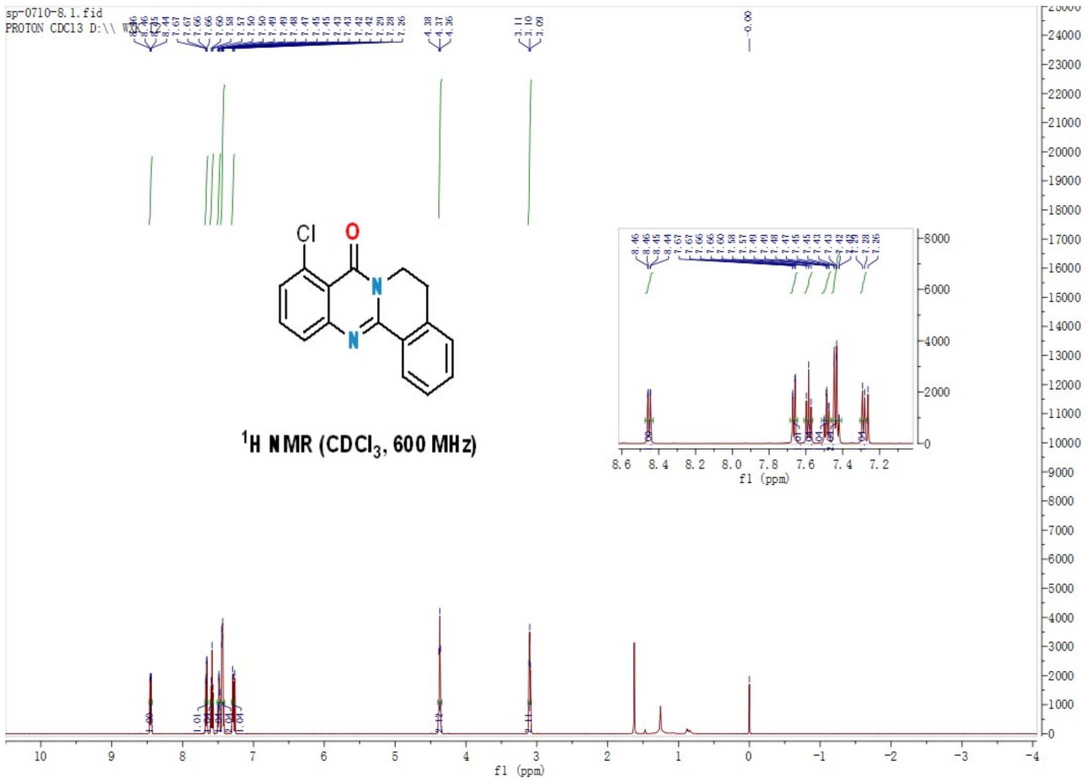
sp-0710-8.1.fid
PROTON CDCl3 D:\\ WXK 12
Cl
O
N
N
1H NMR (CDCl3, 600 MHz)
f1 (ppm)

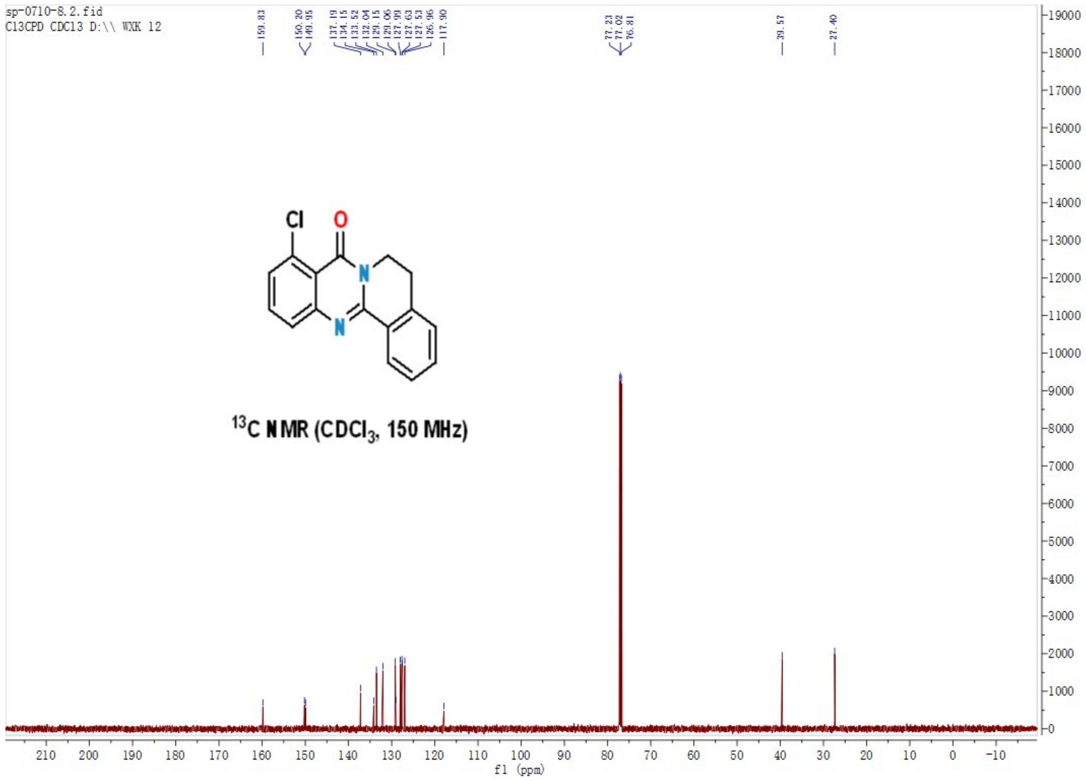
sp-0710-8.2.fid
C13CPD CDCl3 D:\\ WXK 12
159.83
150.20
149.95
137.19
134.15
132.52
132.04
129.15
129.06
127.99
127.63
127.53
126.96
117.90
77.23
77.02
76.81
39.57
27.40
Cl
O
N
N
13C NMR (CDCl3, 150 MHz)
f1 (ppm)

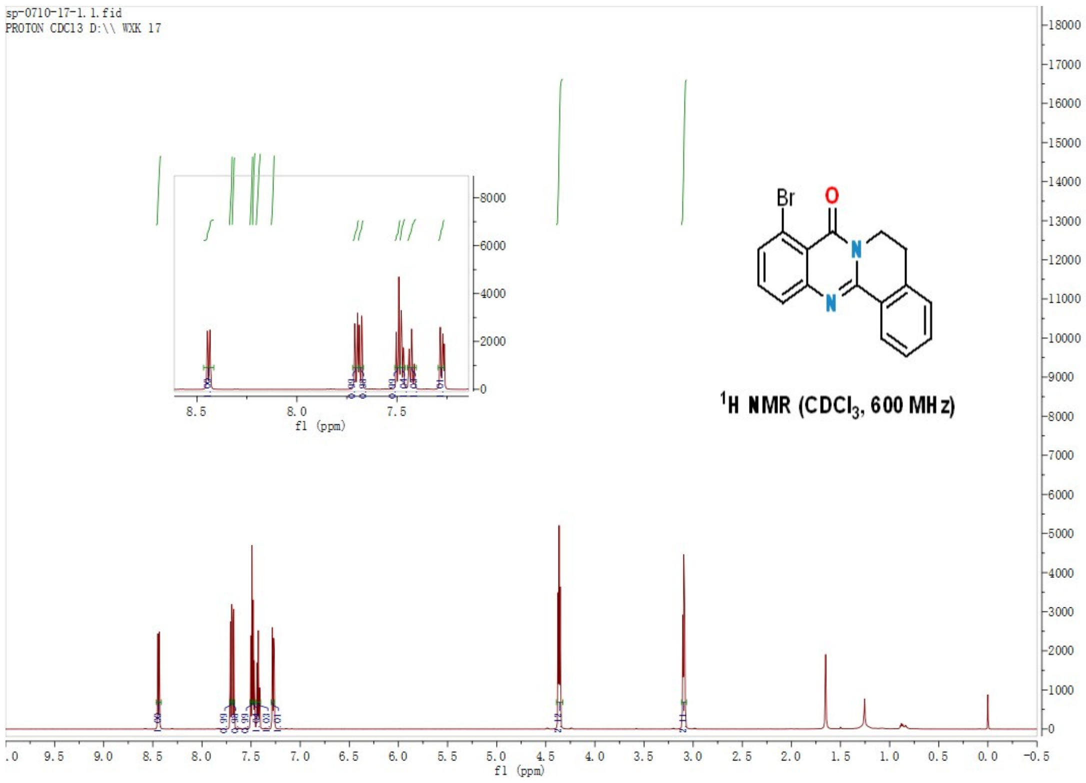
sp-0710-17-1.1.fid
PROTON CDCl3 D:\\ WXK 17
Br
O
N
N
^{1}H NMR (CDCl$_3$, 600 MHz)
f1 (ppm)

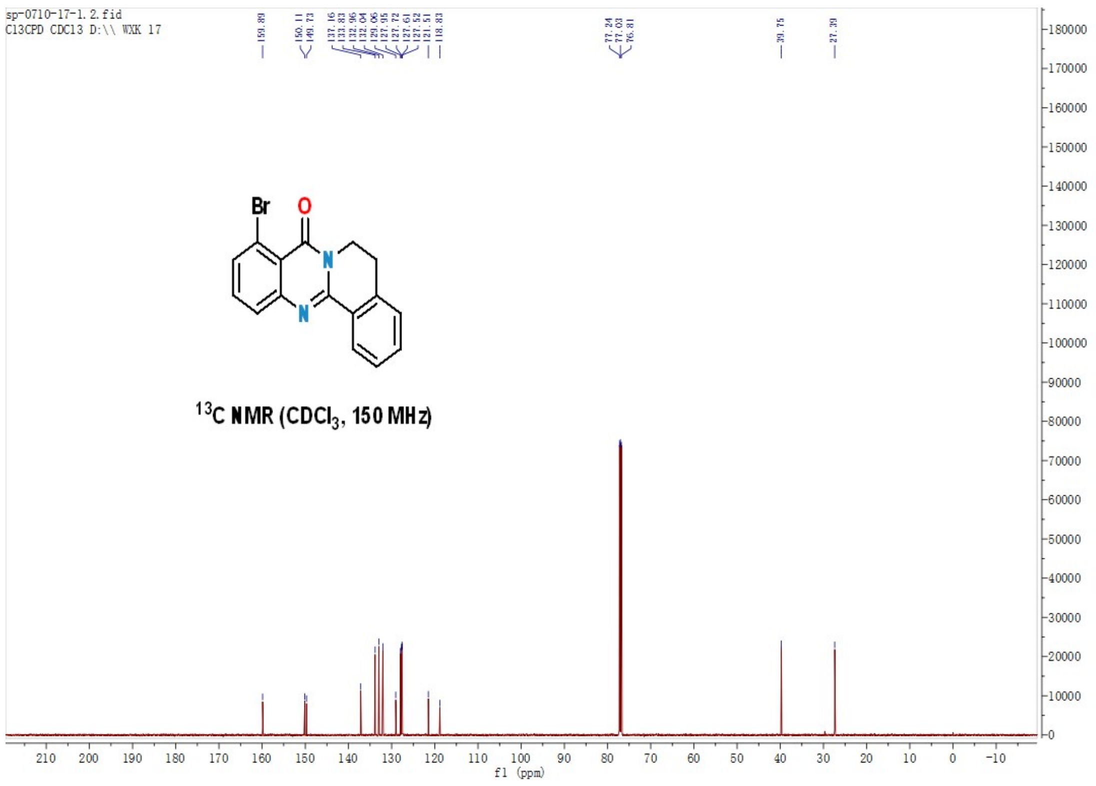
sp-0710-17-1.2.fid
C13CPD CDCl3 D:\\ WXK 17
159.89
150.11
149.73
137.16
133.83
132.96
132.04
129.06
127.95
127.72
127.61
127.52
121.51
118.83
77.24
77.03
76.81
39.75
27.39
Br
O
N
N
^{13}C NMR (CDCl$_3$, 150 MHz)
f1 (ppm)

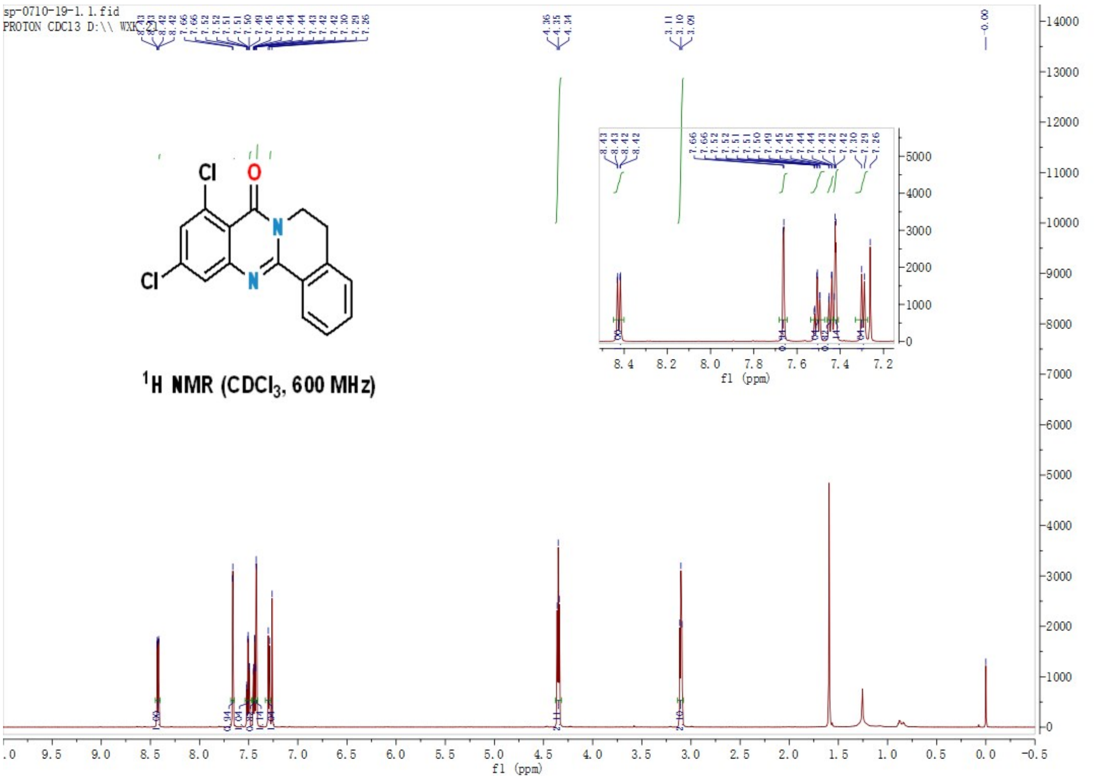
sp-0710-19-1.1.fid
PROTON CDCl3 D:\\ WXK 21
Cl
O
N
Cl
N
1H NMR (CDCl3, 600 MHz)
f1 (ppm)
f1 (ppm)

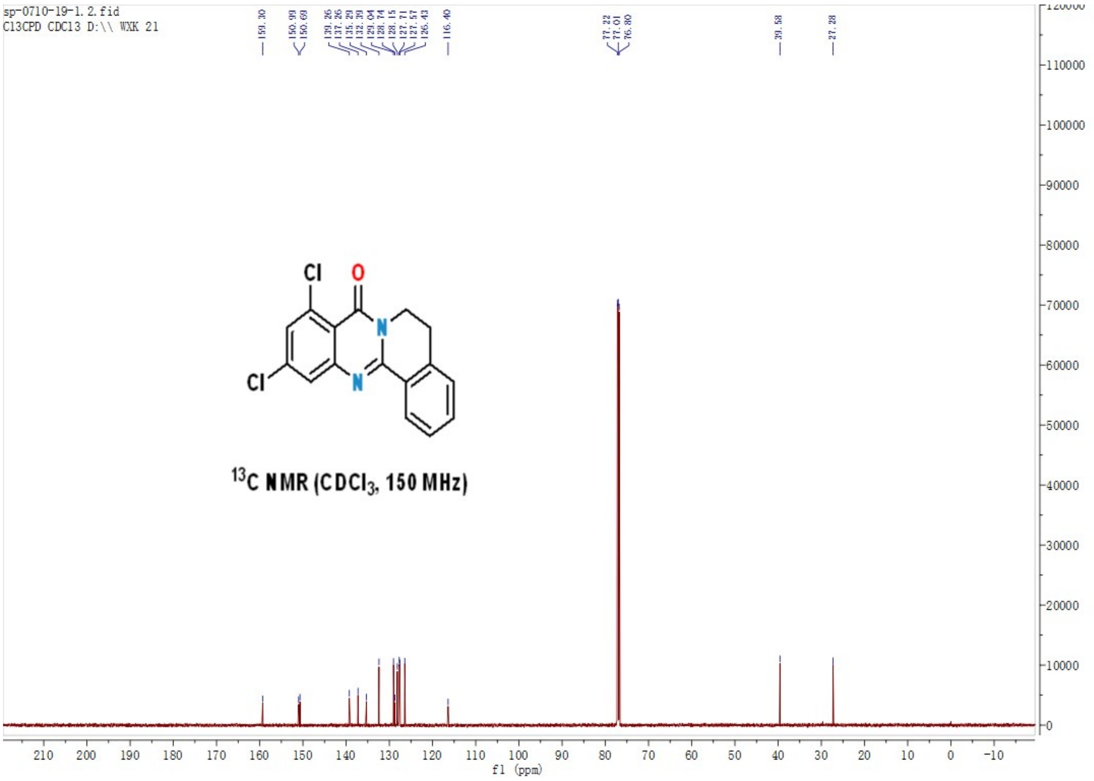
sp-0710-19-1.2.fid
C13CPD CDCl3 D:\\ WXK 21
159.30
150.99
150.69
116.40
77.22
77.01
76.80
39.58
27.28
Cl
O
N
Cl
N
13C NMR (CDCl3, 150 MHz)
f1 (ppm)

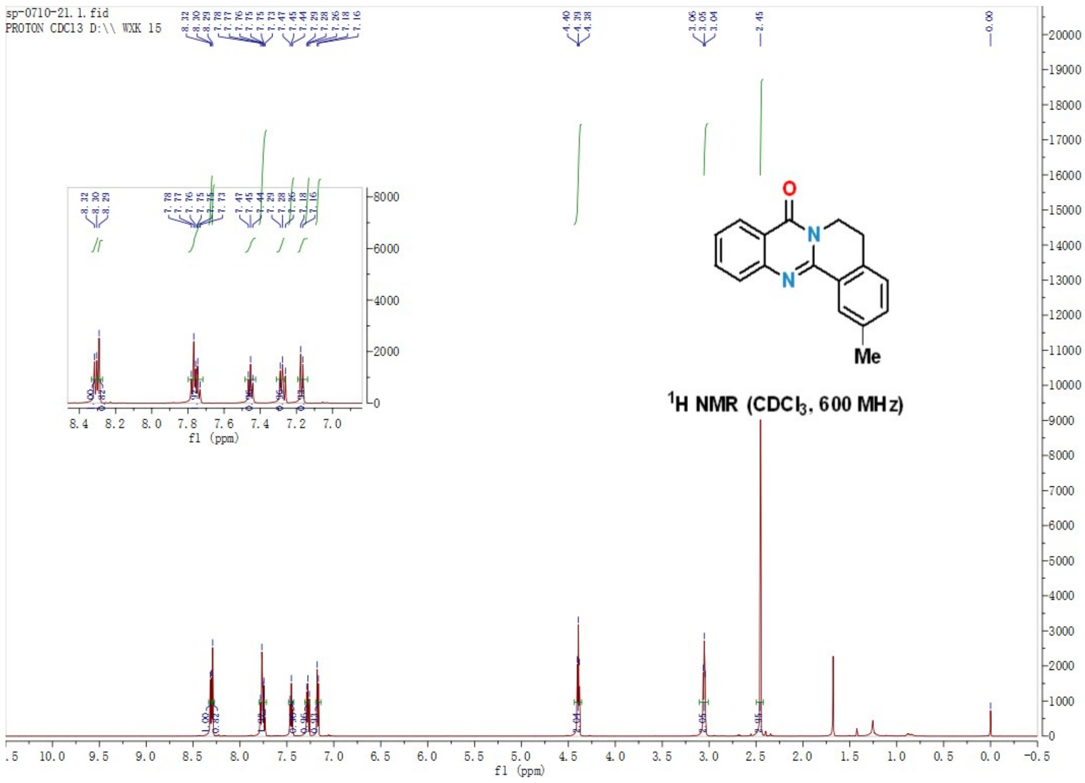
sp-0710-21.1.fid
PROTON CDCl3 D:\\ WXK 15
Me
1H NMR (CDCl3, 600 MHz)
f1 (ppm)

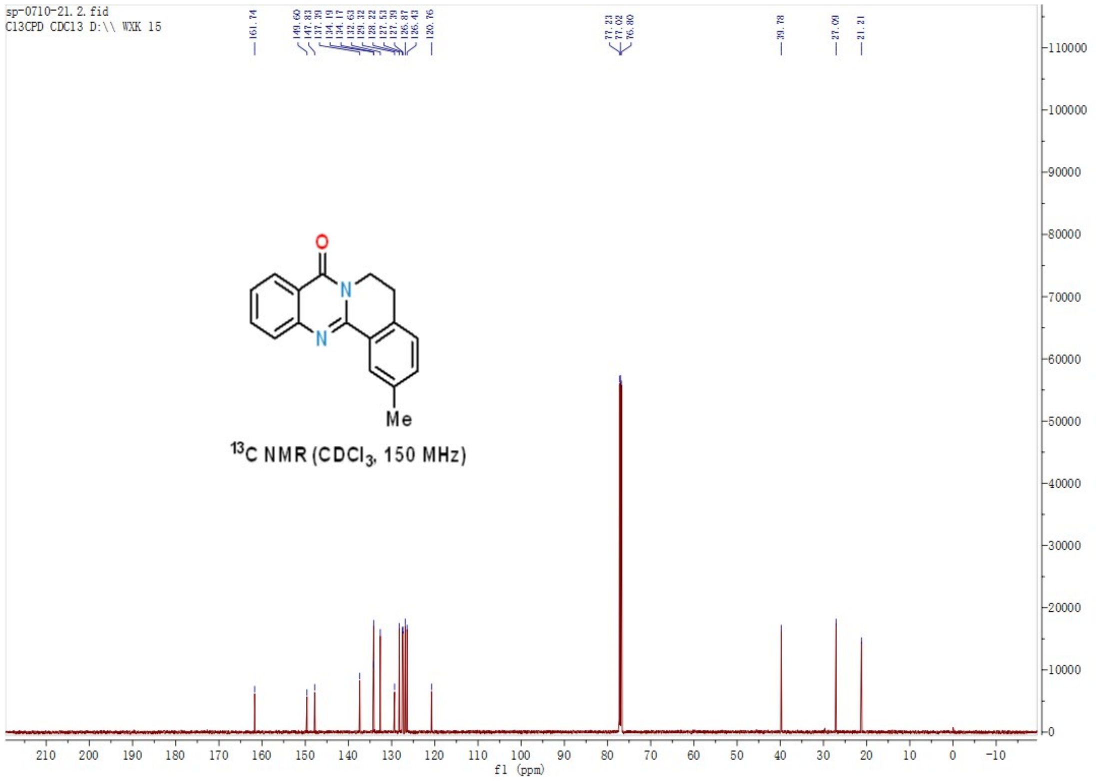
sp-0710-21.2.fid
C13CPD CDCl3 D:\\ WXK 15
Me
13C NMR (CDCl3, 150 MHz)
f1 (ppm)

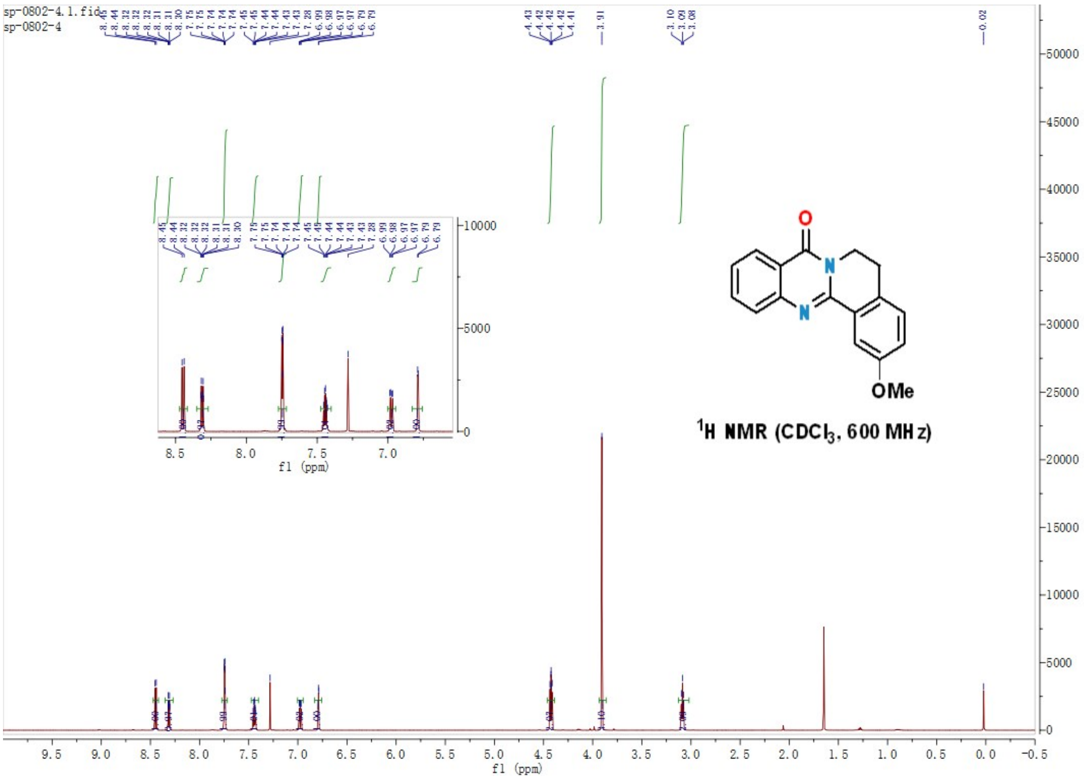
sp-0802-4.1.fid
sp-0802-4
OMe
1H NMR (CDCl3, 600 MHz)
f1 (ppm)

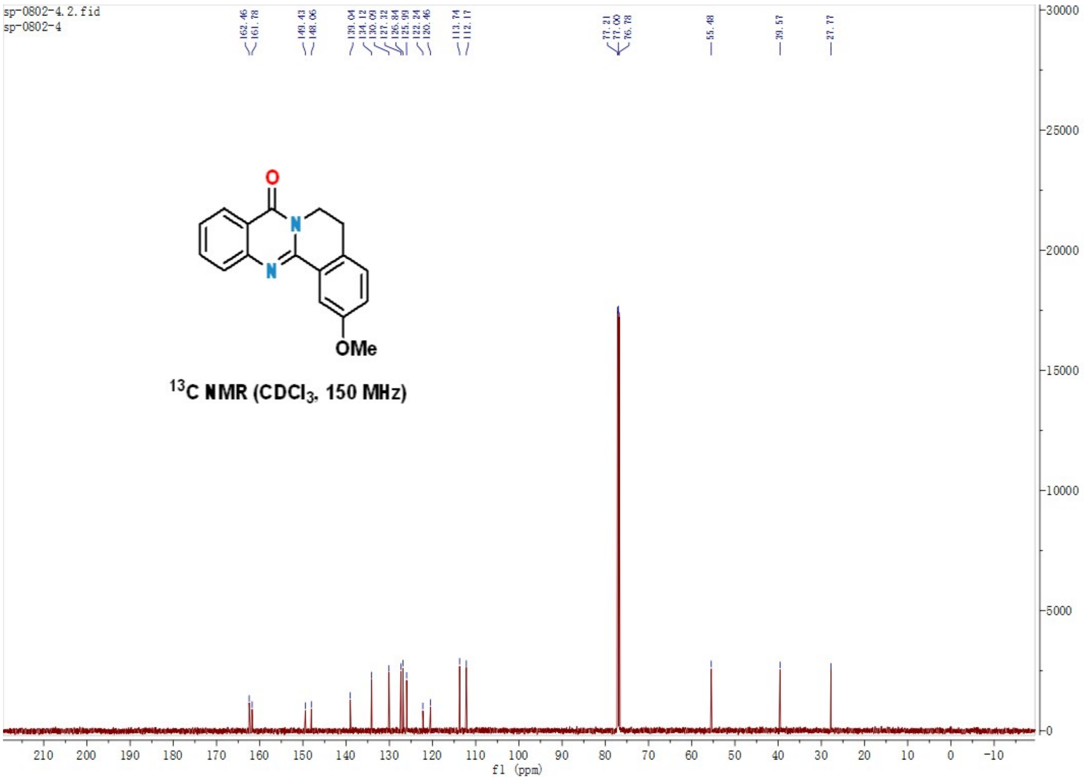
sp-0802-4.2.fid
sp-0802-4
162.46
161.78
149.43
148.06
139.04
134.12
130.09
127.32
126.84
125.99
122.24
120.46
113.74
112.17
77.21
77.00
76.78
55.48
39.57
27.77
OMe
13C NMR (CDCl3, 150 MHz)
f1 (ppm)

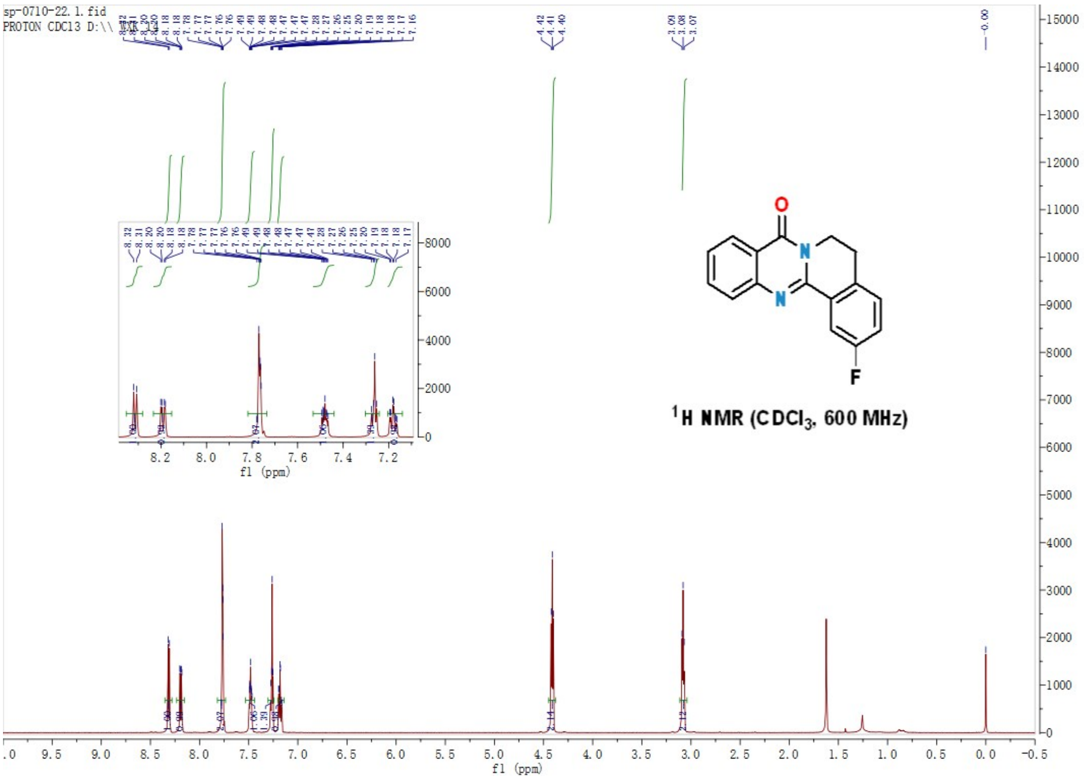
sp-0710-22.1.fid
PROTON CDCl3 D:\\ WXK 14
^{1}H NMR (CDCl$_3$, 600 MHz)
f1 (ppm)

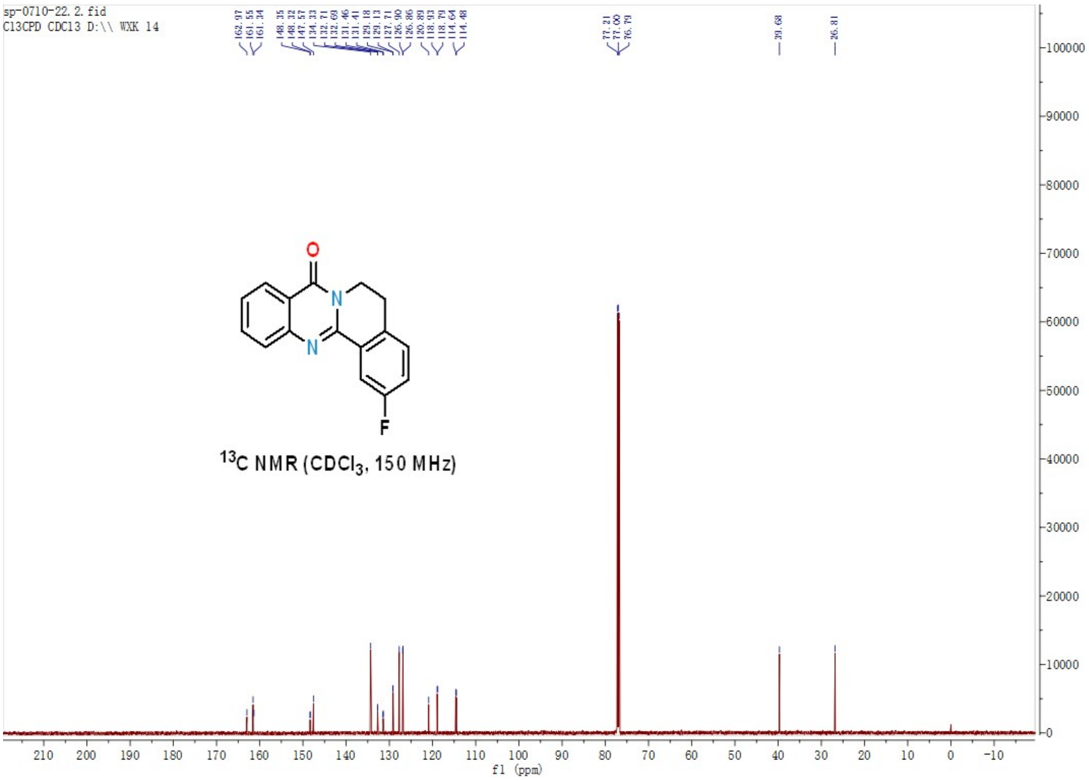
sp-0710-22.2.fid
C13CPD CDCl3 D:\\ WXK 14
^{13}C NMR (CDCl$_3$, 150 MHz)
f1 (ppm)

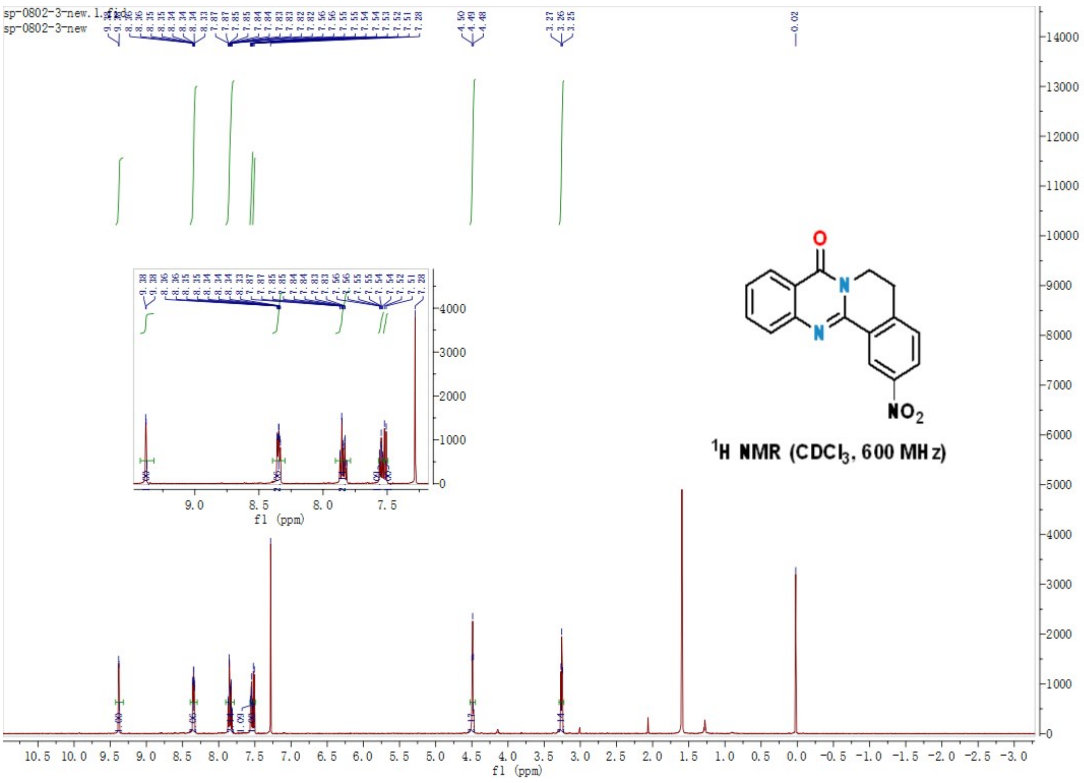
sp-0802-3-new.1.fid
sp-0802-3-new
^{1}H NMR (CDCl$_3$, 600 MHz)
NO$_2$
f1 (ppm)

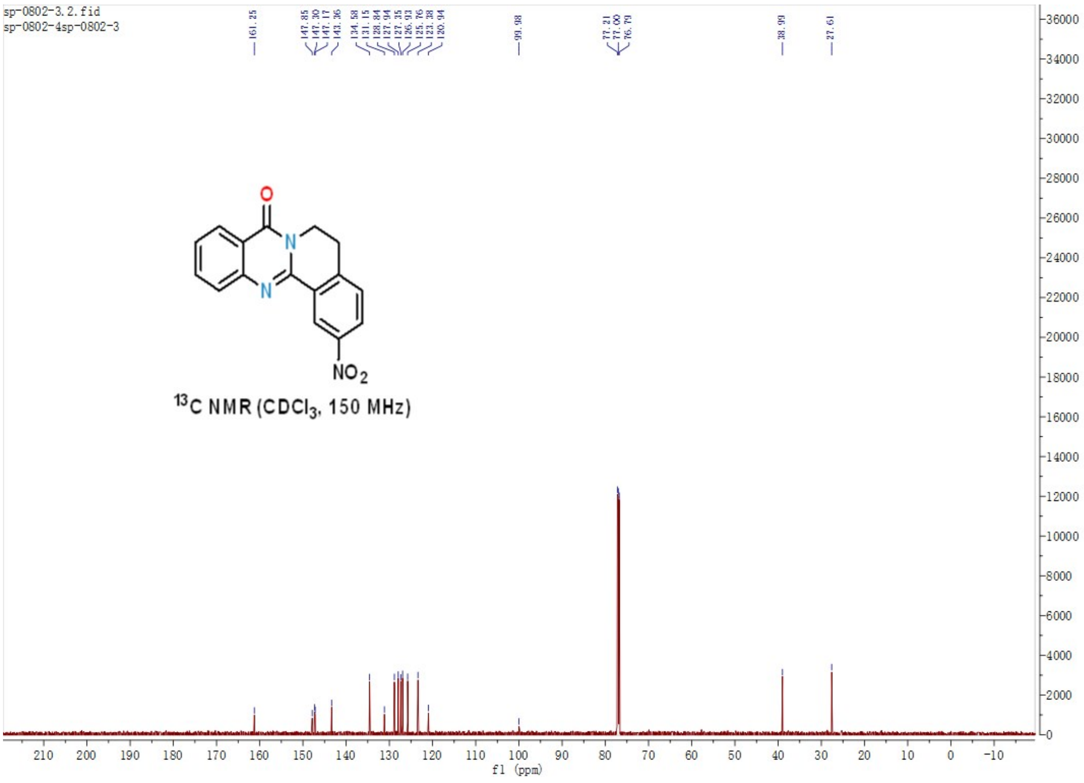
sp-0802-3.2.fid
sp-0802-4sp-0802-3
^{13}C NMR (CDCl$_3$, 150 MHz)
NO$_2$
f1 (ppm)

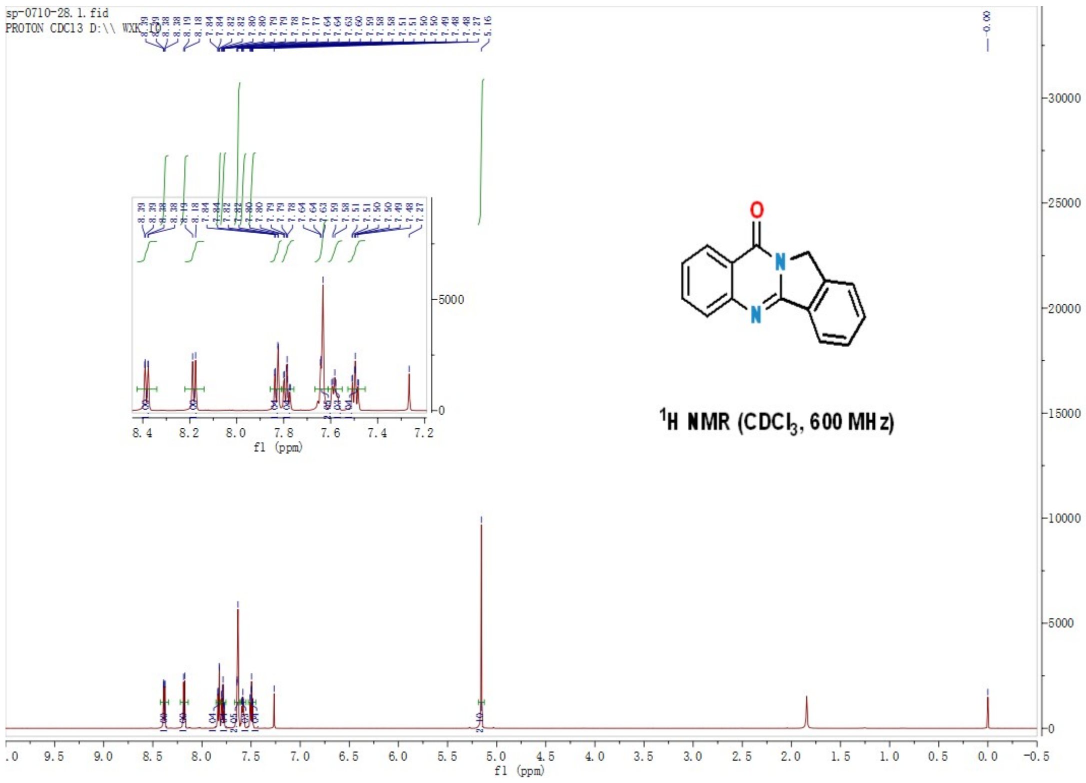
sp-0710-28.1.fid
PROTON CDCl3 D:\\ WXK 10
1H NMR (CDCl3, 600 MHz)
f1 (ppm)

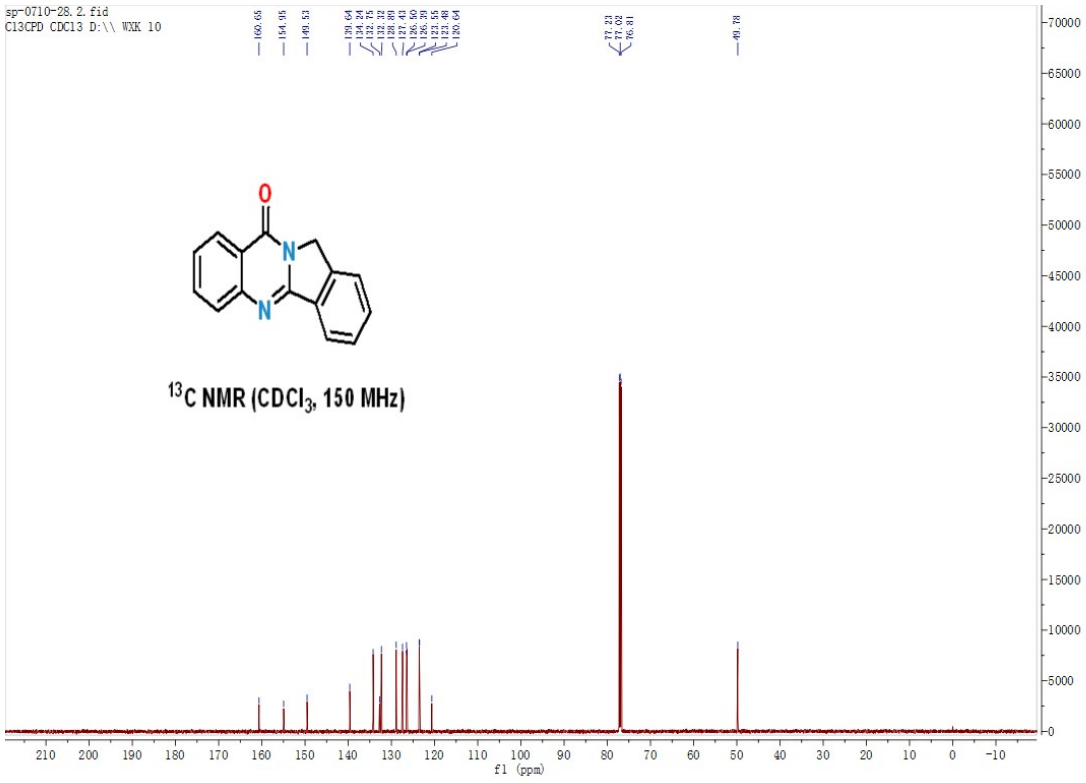
sp-0710-28.2.fid
C13CPD CDCl3 D:\\ WXK 10
160.65
154.95
149.53
139.64
134.24
132.75
132.32
128.89
127.43
126.50
126.39
121.55
123.48
120.64
77.23
77.02
76.81
49.78
13C NMR (CDCl3, 150 MHz)
f1 (ppm)

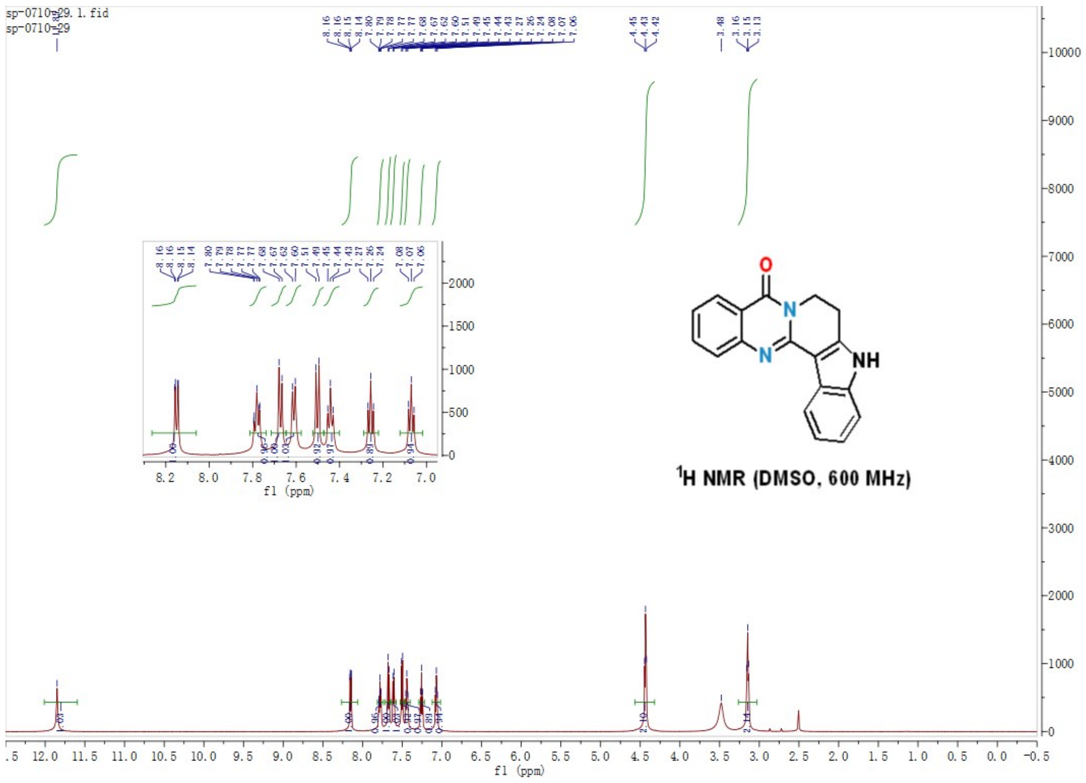
sp-0710-29.1.fid
sp-0710-29
1H NMR (DMSO, 600 MHz)
f1 (ppm)

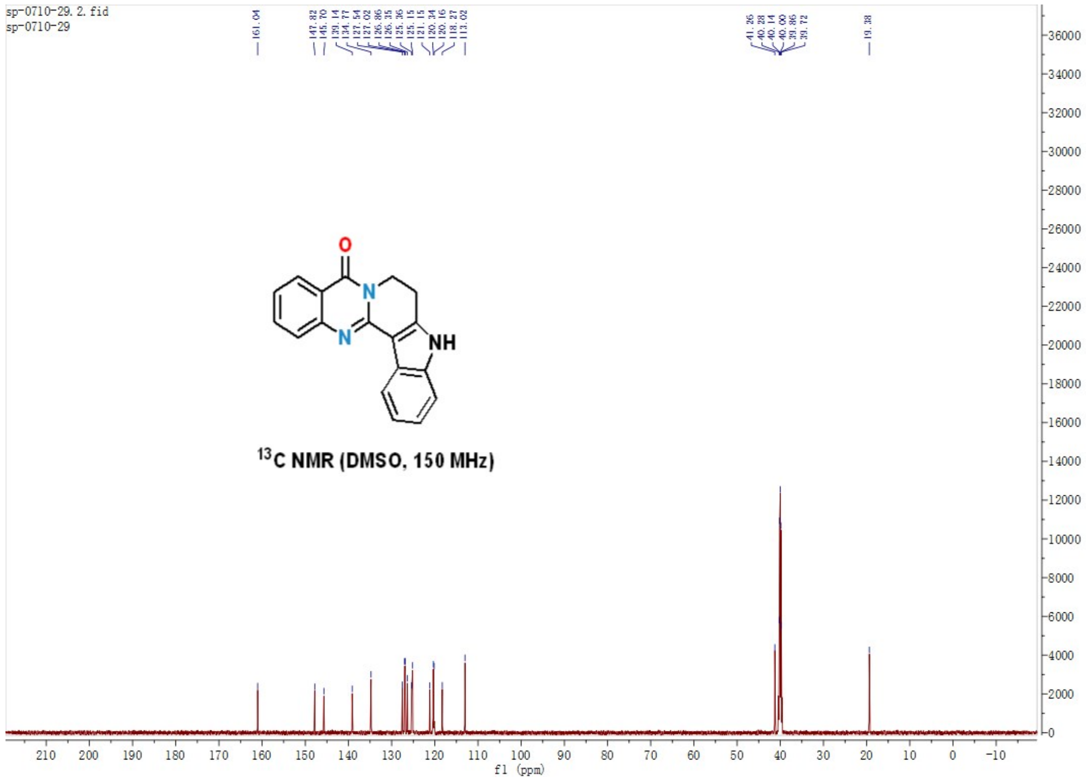
sp-0710-29.2.fid
sp-0710-29
161.04
147.82
145.70
139.14
134.77
127.54
127.02
126.86
126.35
125.36
125.15
121.15
120.34
120.16
118.27
113.02
41.26
40.28
40.14
40.00
39.86
39.72
19.38
13C NMR (DMSO, 150 MHz)
f1 (ppm)

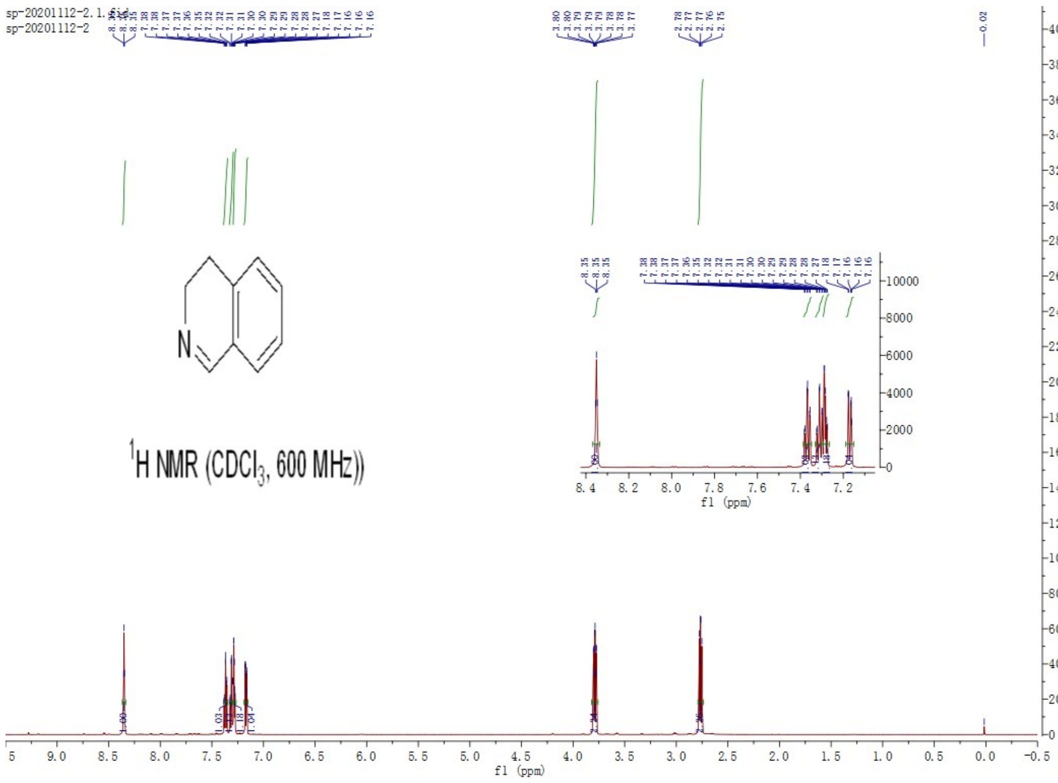
sp-20201112-2.1.fid
sp-20201112-2
N
1H NMR (CDCl3, 600 MHz))
f1 (ppm)

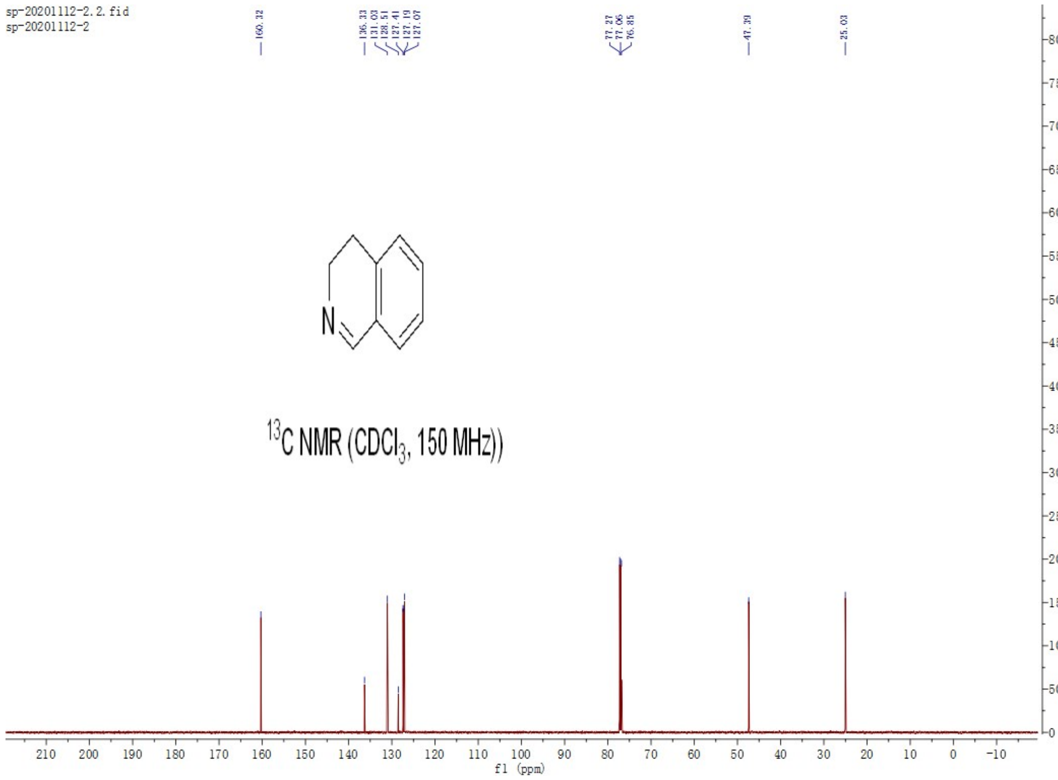
sp-20201112-2.2.fid
sp-20201112-2
160.32
136.33
131.03
128.51
127.41
127.19
127.07
77.27
77.06
76.85
47.39
25.03
N
13C NMR (CDCl3, 150 MHz))
f1 (ppm)

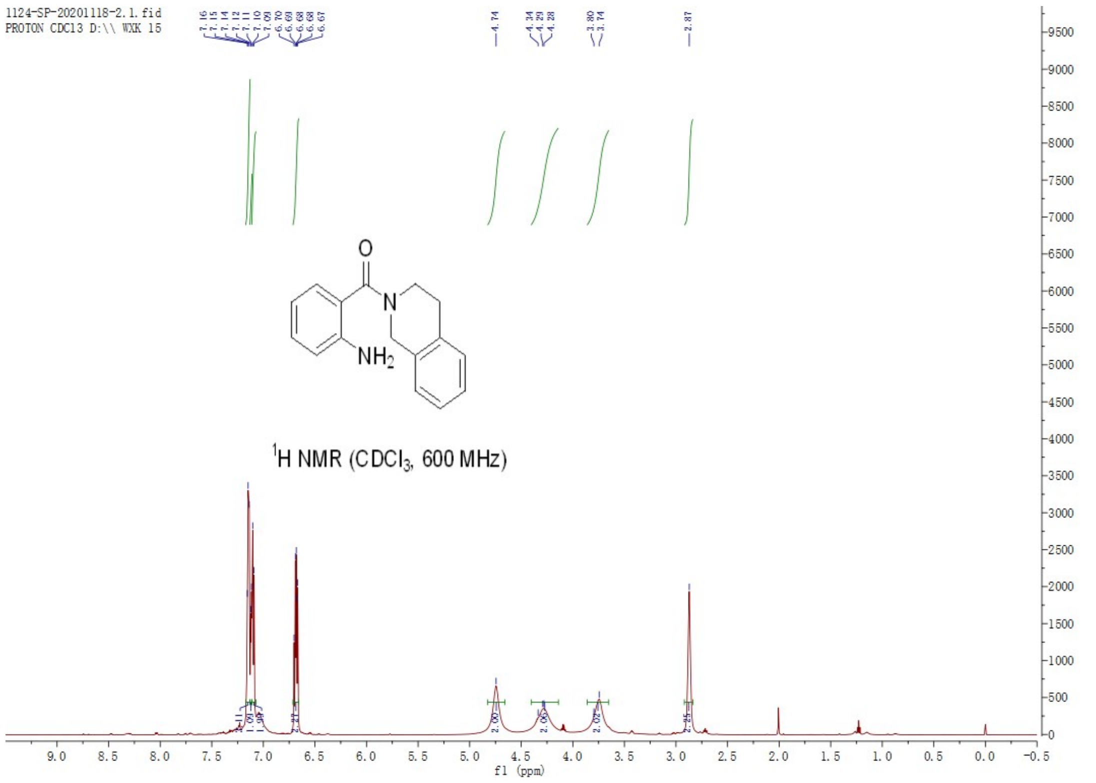

^1H NMR ($CDCl_3$, 600 MHz)

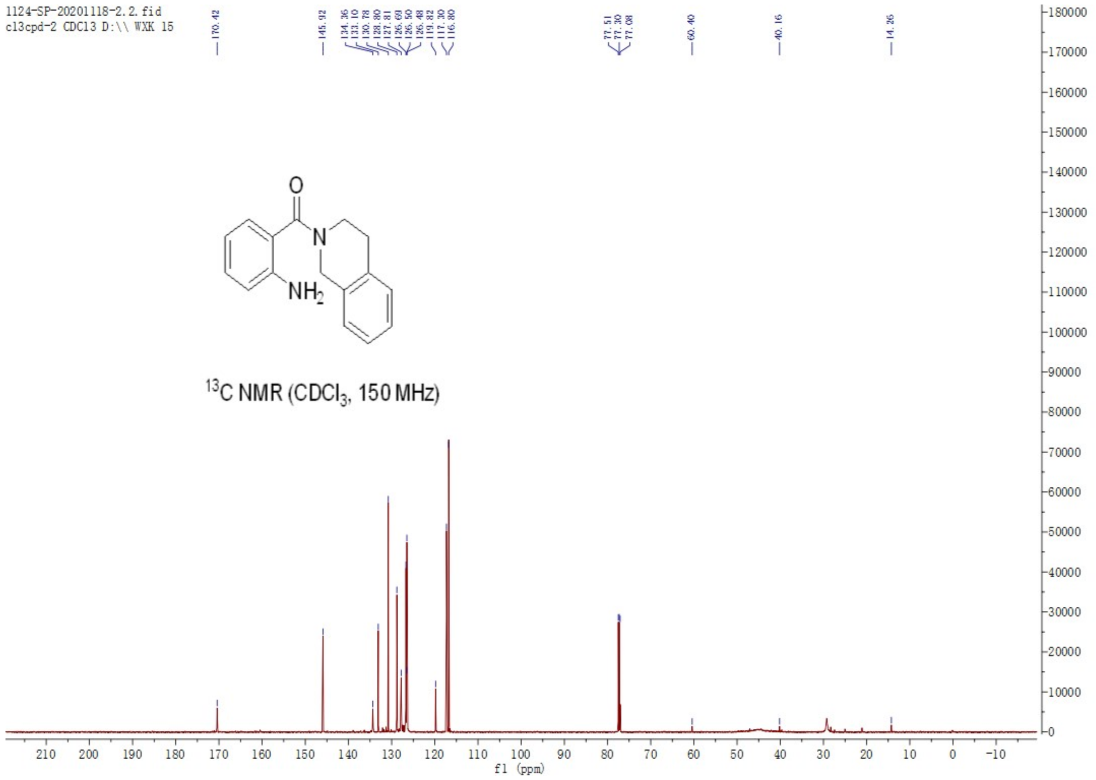

^{13}C NMR ($CDCl_3$, 150 MHz)
